# A unified population pharmacokinetic model of amikacin in paediatric patients from birth to 18 years for model-informed precision dosing

**DOI:** 10.1093/jac/dkag354

**Published:** 2026-10-22

**Authors:** Najla Alabdulkarim, Charlotte Barker, Bairavi Indrakumar, James Hatcher, Orlagh McGarrity, Timothy Best, Iek Leng Cheng, Joseph F Standing

**Affiliations:** Great Ormond Street Institute of Child Health, University College London, London, UK; Department of Clinical Pharmacy, Princess Nourah bint Abdulrahman University, Riyadh, Saudi Arabia; Great Ormond Street Institute of Child Health, University College London, London, UK; Department of Pharmacy, Great Ormond Street Hospital for Children, London, UK; Department of Microbiology, Great Ormond Street Hospital for Children, London, UK; Department of Pharmacy, Great Ormond Street Hospital for Children, London, UK; Department of Pharmacy, Great Ormond Street Hospital for Children, London, UK; Department of Pharmacy, Great Ormond Street Hospital for Children, London, UK; Great Ormond Street Institute of Child Health, University College London, London, UK; Department of Pharmacy, Great Ormond Street Hospital for Children, London, UK

## Abstract

**Background:**

Amikacin is widely used in paediatric patients, but developmental changes in drug disposition from birth through to adolescence make dose optimization challenging. Existing population pharmacokinetic (PopPK) models are typically developed for specific paediatric age groups, limiting their applicability across the full paediatric age range.

**Objectives:**

To develop a unified paediatric PopPK model of amikacin capable of predicting drug concentrations from birth to 18 years of age and to evaluate its suitability for model-informed dosing.

**Methods:**

Retrospective therapeutic drug monitoring data were obtained from 409 paediatric patients receiving intravenous amikacin at a tertiary children's hospital. A PopPK model was developed using data from 204 neonates, which incorporated mechanistic covariates describing growth and renal maturation, including allometric body-weight scaling, postmenstrual age (PMA), postnatal age (PNA) and age-adjusted serum creatinine. Predictive performance was evaluated for 205 older patients aged 1 month to 18 years, and compared with previously published paediatric amikacin PopPK models. The model developed using the neonatal data was subsequently refitted to the combined dataset to obtain the final parameter estimates.

**Results:**

A two-compartment model adequately described amikacin pharmacokinetics. Clearance was influenced by body weight, PMA, PNA, age-adjusted serum creatinine and concomitant glycopeptide use. The model developed in neonates using mechanistic maturation and renal function covariates successfully predicted amikacin concentrations in older paediatric patients and showed lower prediction bias and improved precision than previously published paediatric amikacin models. The final parameter estimates were obtained by fitting the model developed on neonates to all patients.

**Conclusions:**

A maturation-informed PopPK model successfully predicted amikacin concentrations from birth to 18 years and provides a unified framework for model-informed precision dosing across the paediatric age range.

## Background

Amikacin remains a key aminoglycoside for the treatment of severe Gram-negative infections in children, particularly in settings with high resistance to other aminoglycosides and in critically ill patients.^1^ Early initiation of appropriate antimicrobial therapy at optimal exposure is essential to reduce morbidity and mortality in this vulnerable population.

Aminoglycosides exhibit concentration-dependent bactericidal activity and a post-antibiotic effect, making high peak concentrations important for efficacy.^2–6^ However, they have a narrow therapeutic index, and excessive exposure is associated with nephrotoxicity and ototoxicity.^2,3,5,7^ To balance efficacy and safety, treatment typically begins with a standard initial dose, followed by therapeutic drug monitoring (TDM) to guide subsequent dose adjustments.^8,9^ As dose adjustment depends on measured concentrations obtained after treatment initiation, the first doses are typically empirical and early exposure may be suboptimal.

The pharmacokinetics (PK) of amikacin in children are characterized by substantial inter-individual variability (IIV), largely driven by developmental changes in body composition and renal function.^10–12^ Renal clearance is the primary elimination pathway and undergoes rapid maturation during early life. Estimation of renal function in neonates and young children remains challenging, as serum creatinine (Scr) is influenced by maternal creatinine levels in the newborn period and also by physiological maturation.^13–15^ These developmental factors complicate covariate modelling of clearance across the paediatric age spectrum. Consequently, standard dosing may result in suboptimal exposure, particularly in younger patients with rapidly changing renal function.

Population pharmacokinetic (PopPK) modelling provides a framework for describing variability in drug disposition and for supporting model-informed precision dosing (MIPD). Several PopPK models of amikacin have been published in paediatric populations.^16–19^ However, most focus on specific age groups, particularly neonates, and use heterogeneous covariate and scaling approaches. Consequently, dosing recommendations often rely on age-specific models rather than a single unified framework, contributing to variability across guidelines.

Standardized, biologically informed approaches incorporating allometric scaling and maturation functions have been proposed for renally eliminated drugs and applied to aminoglycosides such as gentamicin and other renally cleared antibiotics, supporting PK modelling across the paediatric age range.^20–23^ Building on these mechanistic principles, we hypothesized that, since the greatest PK variability occurs during the neonatal period, where postmenstrual age (PMA), postnatal age (PNA), body weight and Scr change rapidly, a mechanistic model incorporating biologically informed covariates describing body size and renal maturation would also be predictive of the PK in older children.^23,24^ After testing this hypothesis to arrive at an optimal covariate parameterization, we aimed to develop a unified maturation-informed PopPK model of amikacin applicable from extremely preterm neonates to 18 years of age, to support model-informed initial dosing in paediatric patients.

## Method

### Study population

This study used clinical data obtained retrospectively from patients admitted to Great Ormond Street Hospital for Children, UK, between September 2020 and December 2022. Eligible patients were aged ≤18 years and received intravenous amikacin for at least 3 days.

The model-building dataset consisted of neonatal patients (birth to <1 month), while the evaluation dataset included older paediatric patients (1 month–18 years). Following external evaluation, the datasets were combined for final model parameter estimation.

The study was approved by the London and South East Research Ethics Committee (reference 21/LO/0646). As anonymized retrospective data were used, the requirement for informed consent was waived.

### Amikacin dosing and sampling

Intravenous amikacin therapy was initiated at the discretion of the clinical team according to hospital policy dosing guidelines: 10–15 mg/kg once daily for neonates, 20 mg/kg once daily for infants, children and adolescents, 30 mg/kg once daily in children with cystic fibrosis, 10 mg/kg in patients with renal impairment, with dosing intervals guided by TDM. Doses ≥20 mg/kg were administered as 30-min intravenous infusions, whereas lower doses were administered as slow bolus injections over approximately 3 min. Serum amikacin concentrations were obtained as part of routine TDM. Total amikacin concentrations were quantified using a Quantitative Microsphere System homogeneous particle-enhanced turbidimetric immunoassay on the Indiko Plus analyser, with an assay range of 0.8–100 mg/L. Sampling times and dosing histories were recorded for each patient.

### Data collection

Electronic patient records were reviewed retrospectively to extract demographic, clinical and PK data, including age, weight, gestational age, PMA, PNA, Scr concentration, dosing history and concomitant medications. Scr was measured using an enzymatic assay. Gestational age was available for 197/409 patients (48.2%), including 169/204 neonates (82.8%). Where gestational age was unavailable, 40 weeks was assumed for calculation of PMA.

### Data preparation

Patients with fewer than two quantifiable amikacin concentrations were excluded, irrespective of sampling type (peak or trough). Only the first treatment episode per patient was included in the analysis to avoid introducing unmodelled inter-occasion variability between separate treatment courses.

### Software

All data processing and modelling were undertaken with R (version 4.5.3).^25^ The PK analysis was performed using the (nlmixr2) package (version 7.0.1), while visualization and graphical evaluation of model outputs were facilitated by the xpose package (version 0.4.23) and ggplot2 (version 4.0.3).^26,27^

### Pharmacokinetic analysis

The first-order conditional estimation with interaction (FOCEI) method was used. Below-limit-of-quantification data were handled using the M3 method.^28^

The model was first developed using the neonatal dataset, as this population exhibits the most rapid maturation-related changes in renal clearance, and mechanistic covariates were applied that have been shown to predict renally cleared antibiotic PK across age groups.^29,30^ The model developed using the neonatal data was externally evaluated in the older paediatric dataset without parameter re-estimation to assess its predictive performance and ability to scale across the whole paediatric age range. A comparison was also made with other published models. Following external evaluation, the model was refitted to the combined neonatal and older paediatric dataset to obtain final parameter estimates informed by PK observations across the full paediatric age range.

### Model development

#### Structural model

One- and two-compartment structural models for intravenous administration were evaluated. IIV was assumed to follow a log-normal distribution and was tested on clearance (CL), central volume of distribution (V1), intercompartmental clearance (Q) and peripheral volume (V2). Residual unexplained variability was tested using additive, proportional and combined error models.

#### Covariate model

Allometric weight scaling was applied relative to a standard body weight of 70 kg.^31^ Clearance parameters were scaled using a fixed exponent of 0.75, and volumes were scaled with an exponent of 1:


(1) $$F_{siz}=\;{\left(\frac{WT_{i}}{WT_{std}}\right)}^{\beta_{WT}}$$

where WT*_i_* is the weight of the child in kg, WT_std_ is the standard adult weight 70 kg and *β*_WT_ is the fixed exponent.

Three mechanistic covariates were incorporated to describe maturation-related changes in clearance. PMA: A sigmoidal maturation function adapted from Rhodin *et al*. was used to describe nephron development that begins around the ninth week of gestation (Equation 2).^32,33^ The PMA at which 50% of mature renal function is reached (PMA_50_), and the Hill coefficient were fixed to 47.7 weeks and 3.4, respectively.


(2) $$F_{PMA}=\;\frac{PMA_{i}^{Hill}}{PMA_{50}^{\;\;\;Hill}+PMA_{i}^{Hill}}$$

Scr: Age-adjusted typical Scr values were calculated using a second-order fractional polynomial model described by Ceriotti *et al*. (Equations 3 and 4), accounting for maternal and endogenous creatinine contributions.^34^


(3) $$\begin{array}{rl} TSCR & =\;-2.37330-12.91367\times \ln\;(PNA_{years})\\ & \;+23.93581\;\times \;(PNA_{years}{)}^{0.5} \end{array}$$

(4) $$SCR_{\;function}\;={\left(\;\frac{SCR_{i}}{TSCR}\right)}^{\beta_{Scr}}$$

where TSCR is the typical Scr for the individual's age (µmol/L), and *β*_Scr_ is the exponent describing the effect of SCR on clearance.

PNA: An exponential function was included to account for the rapid renal maturation that occurs during the first weeks of life (Equation 5).


(5) $$PNA_{\;function}=hM+(1-hM)\;\times \;(1-e^{-PNA_{i}\;\times \;hN}$$

where is *PNA_i_* postnatal age expressed in days, *hM* is the fraction of clearance at birth (*PNA* = 0 days) and *hN* is the postnatal maturation rate constant.^23^

Concomitant use of glycopeptides was also evaluated as a potential covariate. Covariate selection used forward inclusion (ΔOFV > 6.63, *P* < 0.01) followed by backward elimination (ΔOFV > 10.83, *P* < 0.001).^35^

### Model evaluation

Model adequacy was assessed using goodness-of-fit (GOF) plots, residual diagnostics and visual predictive checks (1000 simulations). Parameter precision was evaluated using standard errors derived from the covariance step. Parameter uncertainty and model stability were further assessed using a non-parametric bootstrap with 1000 replicates.

### Model predictive performance

Predictive performance in the older paediatric dataset was assessed using maximum a posteriori (MAP) post-hoc estimation. Accuracy and precision were quantified using mean prediction error (MPE) and root-mean-squared error (RMSE) (Equations 6 and 7).^36^


(6) $$\mathrm{MPE}=\frac{1}{n}\;\overset{n}{\underset{i=1}{\sum }}\;(P_{i}-O_{i})$$

(7) $$\mathrm{RMSE}=\sqrt{\frac{1}{n}\;\overset{n}{\underset{i=1}{\sum }}\;{\left(P_{i}-O_{i}\right)}^{2}}$$

where *P_i_* is the predicted value (PRED or IPRED), *O_i_* is the observed value (DV) and *n* is the number of observations.

Prediction error (PE) was calculated as observed minus predicted concentration. Relative predictive performance was assessed using median absolute percentage error (MdAPE) and the proportions of predictions within ±20% and ±30% of observed concentrations (F20 and F30). Corresponding metrics were calculated for individual predictions (MdAIPE, IF20 and IF30) using quantifiable observations.

#### Comparison with published models

Previously published paediatric amikacin PopPK models for comparison were identified through a PubMed literature search using terms ‘amikacin’ AND ‘children’ AND ‘pharmacokinetic’. The predictive performance of the model developed using the neonatal data and published paediatric amikacin models was compared by applying each model to the older paediatric dataset and evaluating prediction errors. Published models were implemented using their reported parameter estimates without re-estimation. Predictive performance was compared using population and individual PE distributions, RMSE, MdAPE, F20 and F30.

### Final model parameter estimation

The model developed using the neonatal data was refitted to the combined dataset (birth to 18 years) using FOCEI estimation to obtain final parameter estimates. The final model code is provided in Supplementary Code S1, available as Supplementary data at *JAC* Online.

### Simulation and probability of target attainment

Monte Carlo simulations were performed using the final PopPK model. For each age group and dosing scenario, 500 virtual patients were generated by resampling individuals with replacement from the observed dataset to preserve covariate distributions. Four simulation scenarios were evaluated: (SIM1) dose comparison (5–35 mg/kg once daily); (SIM2) 20 mg/kg administered every 8, 12 or 24 h; (SIM3) 15 mg/kg administered every 8, 12 or 24 h; and (SIM4) 10 mg/kg administered every 8, 12 or 24 h. Concentration–time profiles were simulated over 72 h. Efficacy and safety were assessed over the final dosing interval ending at 72 h (48–72 h for q24h, 60–72 h for q12h and 64–72 h for q8h regimens) using *C*_max_/MIC ≥10 and *C*_min_ <5 mg/L, respectively.^8,37,38^ PTA was then determined across MIC values from 0.125 to 16 mg/L to characterize changes in target attainment with increasing MIC across the simulated dosing regimens.

## Results

### Patient characteristics

A total of 409 paediatric patients were included, comprising 204 in the model-building dataset and 205 in the evaluation dataset. Overall, 290 of 1091 amikacin concentration observations (26.6%) were BLQ. Demographic and clinical characteristics are summarized in Table 1.

**Table 1.** A summary of patient demographics for both the model building and model evaluation datasets

Characteristic	Model building	Model evaluation				Final model
	Neonate	1 month–2 years	3–6 years	7–12 years	13–18 years	Birth—18 years
Total number of patients	204	72	41	52	40	409
Age, years	0.01	0.29	4.1	10	15	0.12
*Range*	(0.003–0.208)	(0.08–2.33)	(3.03–6.27)	(7–12.28)	(13–18)	(0.003–18)
PMA, weeks	39	54	258	565	829	44
*Range*	(24–44)	(44–161)	(196–367)	(406–681)	(720–992)	(24–992)
Dose, mg/kg	15	20	20	18.5	13	15
*Range*	(6–20)	(7–20)	(9–20)	(5–20)	(7–30)	(5–30)
Dosing frequency, n (%)						
Q12h	1 (0.5)	1(1.2)	0	0	2 (5)	4 (1)
Q24h	191 (99.5)	81 (96.4)	41 (100)	51 (98.1)	36 (90)	400 (97.8)
Q36h	0	1 (1.2)	0	1 (1.9)	0	2 (0.5)
Q48h	0	1 (1.2)	0	0	2 (5)	3 (0.7)
TDM samples per patient, n	2	2	2	2	2	2
*Range*	(2–9)	(2–11)	(2–7)	(2–6)	(2–6)	(2–11)
Weight, kg	2.87	7.7	16.7	33.6	48.5	4.73
*Range*	(0.64–6.38)	(3.34–14.8)	(11–29)	(16–89)	(15–143)	(0.6–143)
SCr, µmol/L	48	24.5	32	42.5	56	41
*Range*	(7–233)	(7–552)	(15–84)	(11–259)	(25–770)	(7–770)
Male, n (%)	116 (57%)	34 (47%)	23 (56%)	38 (73%)	17 (42.5%)	228 (55.7%)
Glycopeptide, n (%)	24 (11.8%)	13 (18.1%)	6 (14.6%)	7 (13.5%)	8 (20%)	58 (14.2%)

The model-building dataset comprised neonates (birth to <1 month) and was used for structural model and covariate development. The model-evaluation dataset included patients aged 1 month to 18 years and was used to assess predictive performance without parameter re-estimation. The final model dataset represents the combined cohort used for parameter refitting across the full paediatric age range. Age-group boundaries were birth to <1 month, ≥1 month to <3 years, ≥3 to <7 years, ≥7 to <13 years and ≥13 to <19 years. Data are presented as median (range) unless otherwise stated. TDM samples represent the number of amikacin concentrations available for analysis per patient, including BLQ values. Dosing interval was determined from the recorded dosing and TDM timing information. Serum creatinine was measured using an enzymatic assay, and amikacin concentrations were quantified using an automated immunoassay.

### Model development

Structural model testing showed that a two-compartment model with combined additive and proportional residual error best described the data. The inclusion of IIV on CL, V1, Q and V2 further improved model fit (Table S1).

Stepwise covariate analysis identified body weight, PMA, PNA, age-adjusted Scr and concomitant glycopeptide use as predictors of clearance (Table S2). Allometric scaling of body weight improvement model fit (ΔOFV = −125.88). The addition of maturation functions for PMA and PNA, standardized Scr and glycopeptide use further reduced unexplained variability, resulting in the lowest objective function value (OFV = 2087.954).

### Predictive evaluation

MPE and RMSE were used to quantify prediction bias and precision, respectively. Predictive performance of the model developed using neonatal data and the final model is reported in Table S3. The distributions of population and individual prediction errors for the model developed using the neonatal data and previously published paediatric amikacin models^16–19,39–42^ are presented in Figure 1, with median PE shown as a descriptive summary of each distribution. A summary of evaluated models is provided in Table S4. The model developed using neonatal data showed the lowest population RMSE (6.20 mg/L) and MdAPE (42.55%) among the evaluated models, with 22.22% and 33.99% of predictions within ±20% and ±30% of observed concentrations, respectively. Individual predictions showed an RMSE of 2.46 mg/L and MdAIPE of 18.29%, with 53.27% and 66.34% of predictions within ±20% and ±30% of observed concentrations, respectively (Table S6).

**Figure 1.**
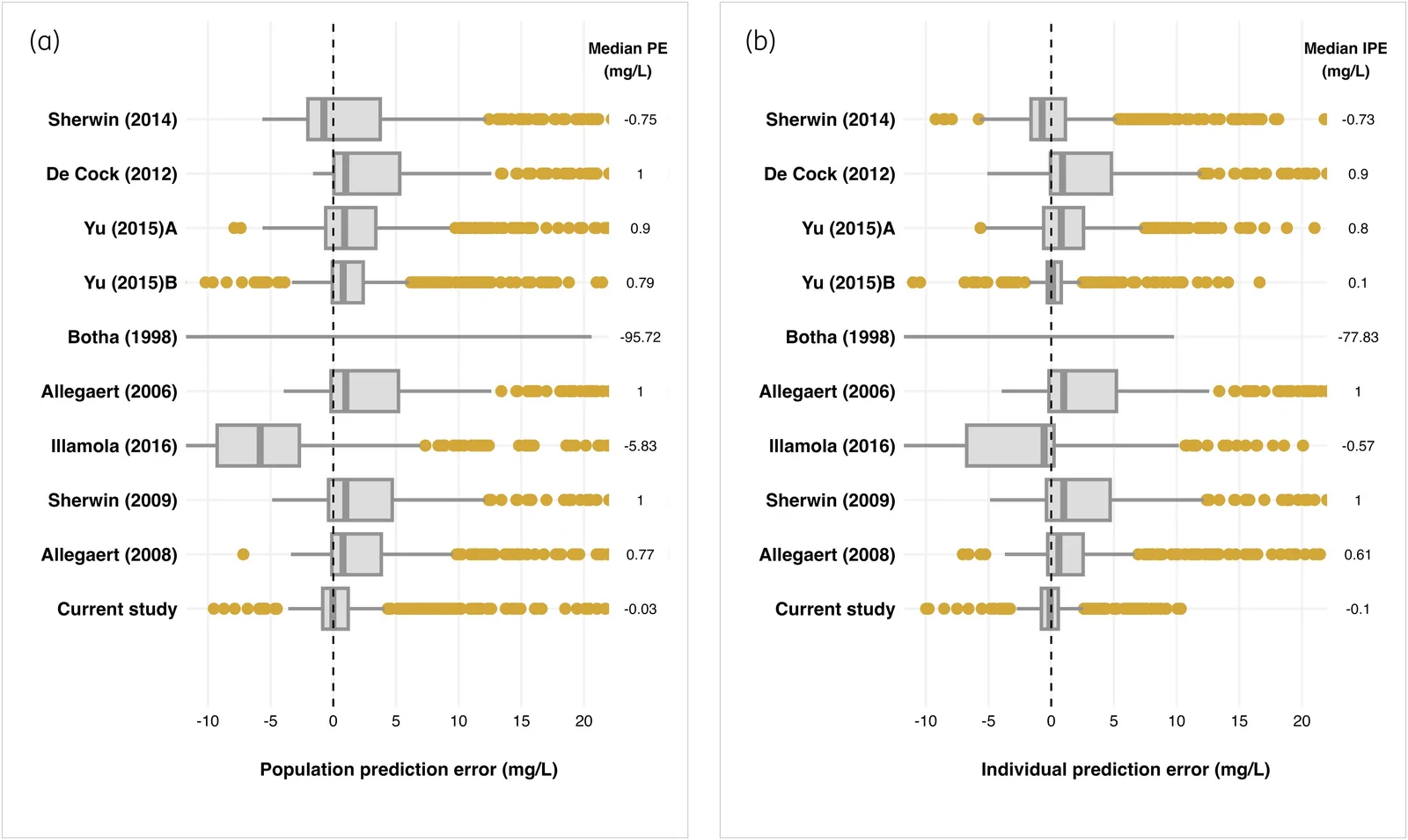
Comparison of prediction errors in the older paediatric dataset for the model developed using the neonatal data and published paediatric amikacin PopPK models. (a) Population prediction error (PRED−OBS). (b) Individual prediction error (IPRED−OBS). Boxplots show the distribution across models; annotated values indicate medians. Boxplots represent the distribution of prediction errors, with the central line indicating the median. Values presented alongside each boxplot represent the median prediction error (PE).

### Final model parameter estimates

Refitting the model to the combined dataset resulted in parameter estimates that were generally comparable to those obtained from the model developed using the neonatal data. The unified model retained a two-compartment structure with clearance influenced by body weight, PMA, PNA, age-adjusted Scr and glycopeptide co-administration. Parameter estimates are presented in Table 2. A 1000-replicate non-parametric bootstrap was performed to further assess parameter uncertainty. A comparison of parameter estimates from the original and refitted models, together with the bootstrap results, is provided in Table S5. Diagnostic plots confirmed adequate model performance (Figure 2), and additional GOF plots are presented in Figure S1.

**Figure 2.**
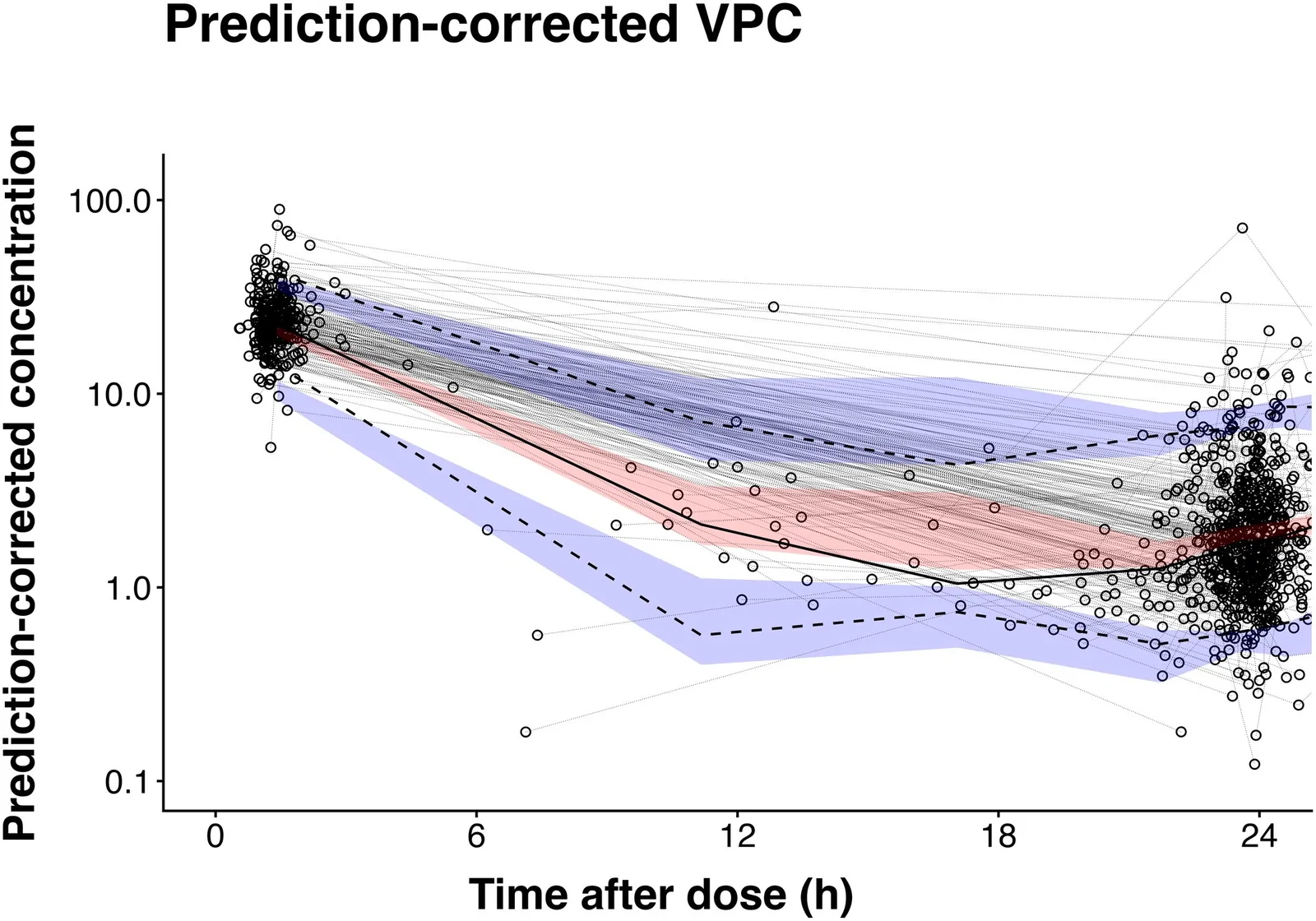
Prediction-corrected visual predictive check (VPC) plot for the final model incorporating both neonatal and paediatric patients. Open circles represent observed concentrations, and dotted lines connect observations from the same individual. The solid line represents the observed median (50th percentile), and the dashed lines represent the observed 5th and 95th percentiles. The shaded region surrounding the median represents its simulation-based 90% confidence interval, and the shaded regions surrounding the 5th and 95th percentiles represent their respective simulation-based 90% confidence intervals.

**Table 2.** The pharmacokinetic parameters of the final model

	Estimate	%RSE	η-shrinkage (%)
Pharmacokinetic parameters			
CL (L/h/70 kg)	5.634	0.20	
V1 (L/70 kg)	29.34	0.16	
Q (L/h/70 kg)	1.07	4.09	
V2 (L/70 kg)	30.26	0.20	
β Scr (CL)	−0.73	0.28	
β glycopeptides (CL)	−0.21	1.55	
hM (CL)	0.44	0.58	
hN (CL)	0.28	0.48	
Between-subject variability			
BSV-CL (CV%)	49.94	—	11.44
BSV-V1 (CV%)	22.79	—	51.39
BSV-Q (CV%)	91.50	—	42.32
BSV-V2 (CV%)	32.70	—	71.25
Residual variability			
Proportional error (%)	24.89	1.71	—
Additive error (mg/L)	1.020	2.42	—

$$TVCL=\;\left[\mathrm{CL}\;\times \;{\left(\frac{WTi}{70}\right)}^{\beta_{WT}}\;\times \;\left(\frac{PMAi^{3.4}}{47.7^{3.4}+PMAi^{3.4}}\right)\;\times \;{\left(\frac{Scri}{TSCR}\right)}^{\beta_{Scr}}\times \;[\mathrm{hM}+(1-\mathrm{hM})\times (1-\exp\;(-PNA\times \mathrm{hN}))]\;\times \;(\mathrm{glycopeptides}{)}^{\beta_{\mathrm{glycopeptide}}}\right]$$

 $$\;TVV1=\;\left[V1\;\times \;{\left(\frac{WTi}{70}\right)}^{\beta_{WT}}\right]$$ $$\;TVQ\;=\;\;\left[Q\;\;\;\times \;{\left(\frac{WTi}{70}\right)}^{\beta_{WT}}\right]$$ $$\;TVV2=\;\left[V2\;\times \;{\left(\frac{WTi}{70}\right)}^{\beta_{WT}}\right]$$

Parameter abbreviations: CL—clearance (L/h); V1—central volume of distribution (L); Q—intercompartmental clearance (L/h); V2—peripheral volume of distribution (L); BSV—between-subject variability, expressed as coefficient of variation (CV%) and calculated as: $\;\sqrt{\exp\;{\left(\omega \right)}^{2}-1\times 100}$

Covariate abbreviations: WT—body weight (scaled to 70 kg); WTi—current body weight; PMA—postmenstrual age function; Scr—age-adjusted mean serum creatinine; PNA—postnatal age; glycopeptides—concomitant use of glycopeptides.

**βWT**—fixed allometric exponent for body weight scaling (0.75 for CL and Q; 1.0 for V1 and V2); β Scr—exponent for age adjusted serum creatinine on CL; β glycopeptides—exponent for glycopeptides use; hM—Initial estimate for fraction of clearance on the first day of life; hN—Initial estimate for postnatal maturation rate constant.

### Probability of target attainment

Across age groups, doses of 15–20 mg/kg A 24 h achieved >95% probability of target attainment (PTA) at an MIC of 2 mg/L. Age-stratified PTA results are summarized in Figures 3–6 and Table S7.

**Figure 3.**
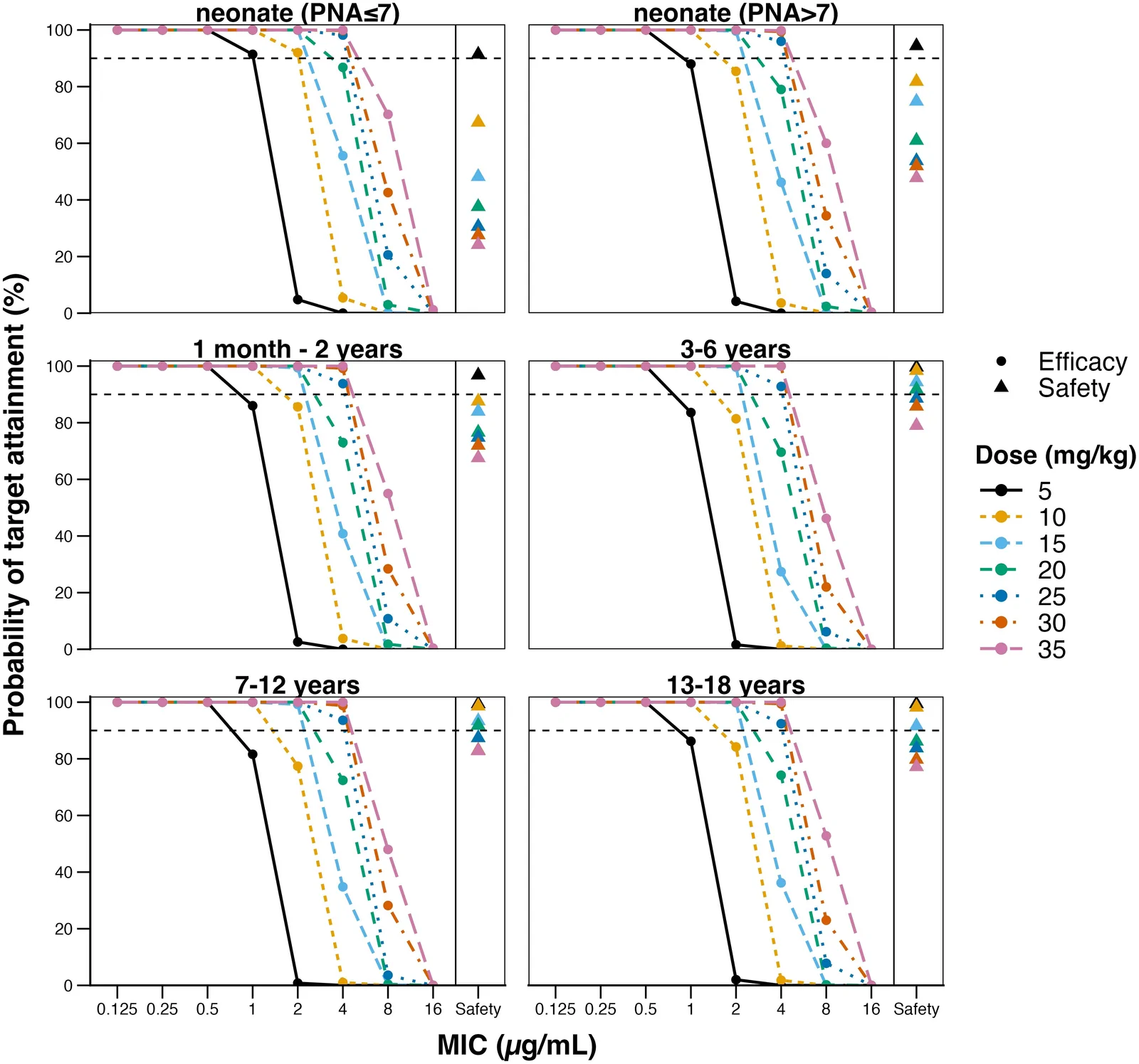
Probability of target attainment (PTA) for efficacy and safety across dose groups (5, 10, 15, 20, 25, 30 and 35 mg/kg) in Simulation 1. Efficacy PTA was defined as the proportion of simulated individuals achieving a peak concentration-to-MIC ratio (*C*_max_/MIC) ≥ 10 across MIC values ranging from 0.125 to 16 mg/L. Safety PTA was defined as the proportion achieving a trough concentration (*C*_min_) below 5 mg/L. The dashed horizontal line represents the 90% PTA target threshold. Results are stratified by age group. Coloured lines indicate different dose groups.

**Figure 4.**
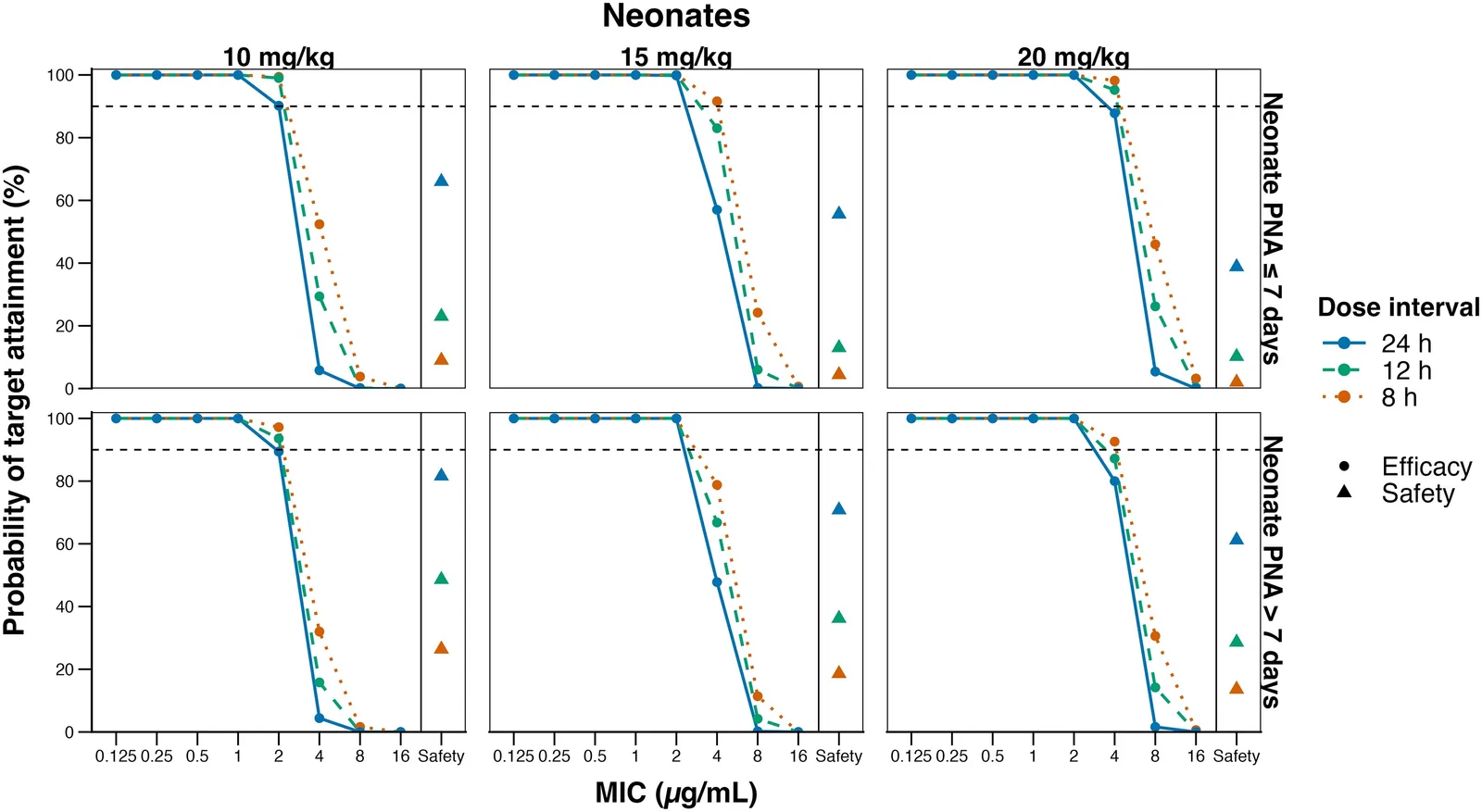
Probability of target attainment (PTA) for efficacy and safety across dosing regimens in neonates. Efficacy PTA was defined as the proportion of simulated individuals achieving a peak concentration-to-MIC ratio (*C*_max_/MIC) ≥ 10 across MIC values from 0.125 to16 mg/L. Safety PTA was defined as the proportion achieving a trough concentration (*C*_min_) < 5 mg/L. The dashed horizontal line represents the 90% efficacy target threshold. Results are shown for dose levels of 10, 15 and 20 mg/kg and stratified by neonatal age group (postnatal age ≤7 and >7 days). Lines represent different dosing intervals (every 24, 12 or 8 hours).

**Figure 5.**
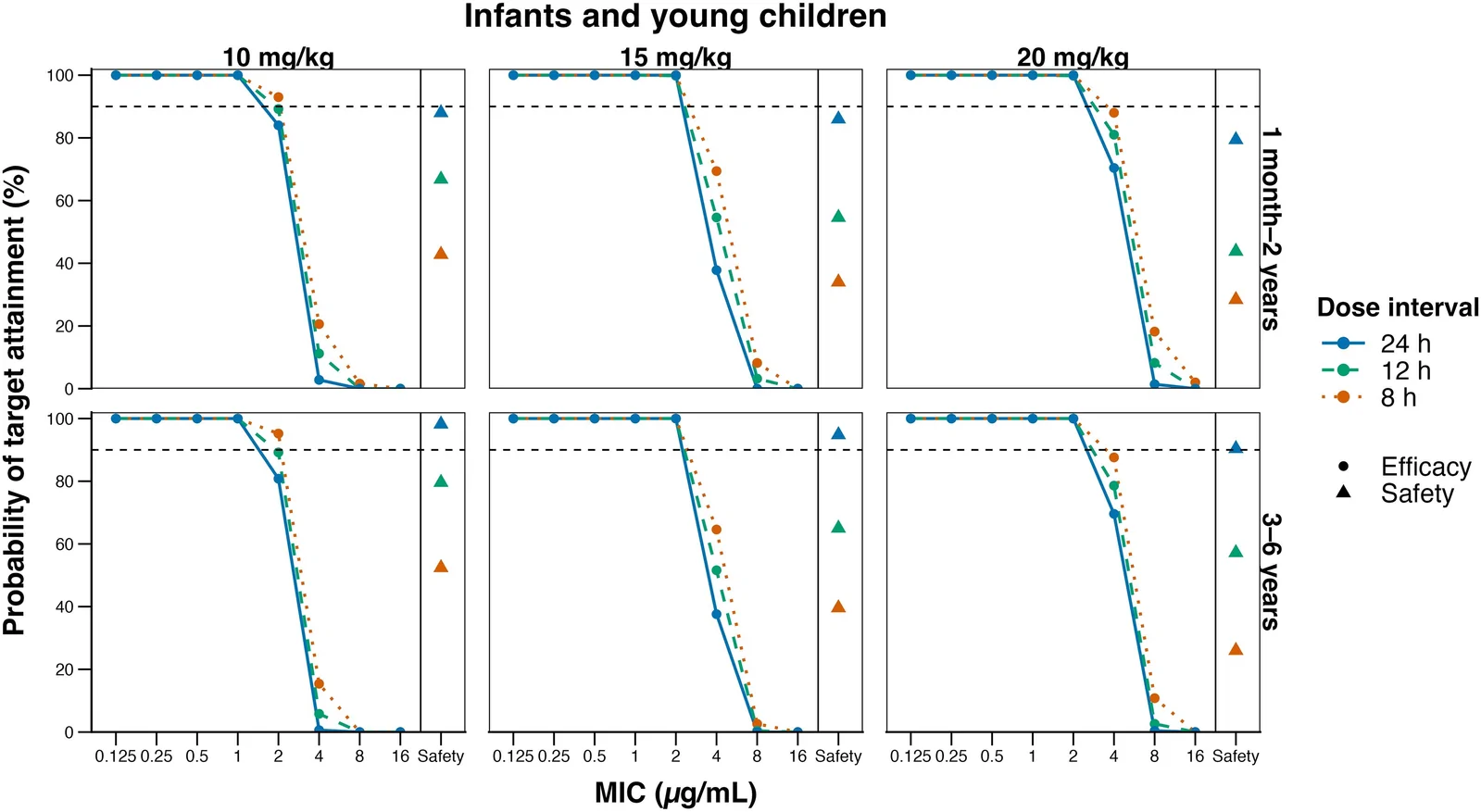
Probability of target attainment (PTA) for efficacy and safety across dosing regimens in infants and young children. Efficacy PTA was defined as the proportion of simulated individuals achieving a peak concentration-to-MIC ratio (*C*_max_/MIC) ≥ 10 across MIC values from 0.125 to 16 mg/L. Safety PTA was defined as the proportion achieving a trough concentration (*C*_min_) < 5 mg/L. The dashed horizontal line represents the 90% efficacy target threshold. Results are shown for dose levels of 10, 15 and 20 mg/kg and stratified by age group (1 month–2 years and 3–6 years). Lines represent different dosing intervals (every 24, 12 or 8 h).

**Figure 6.**
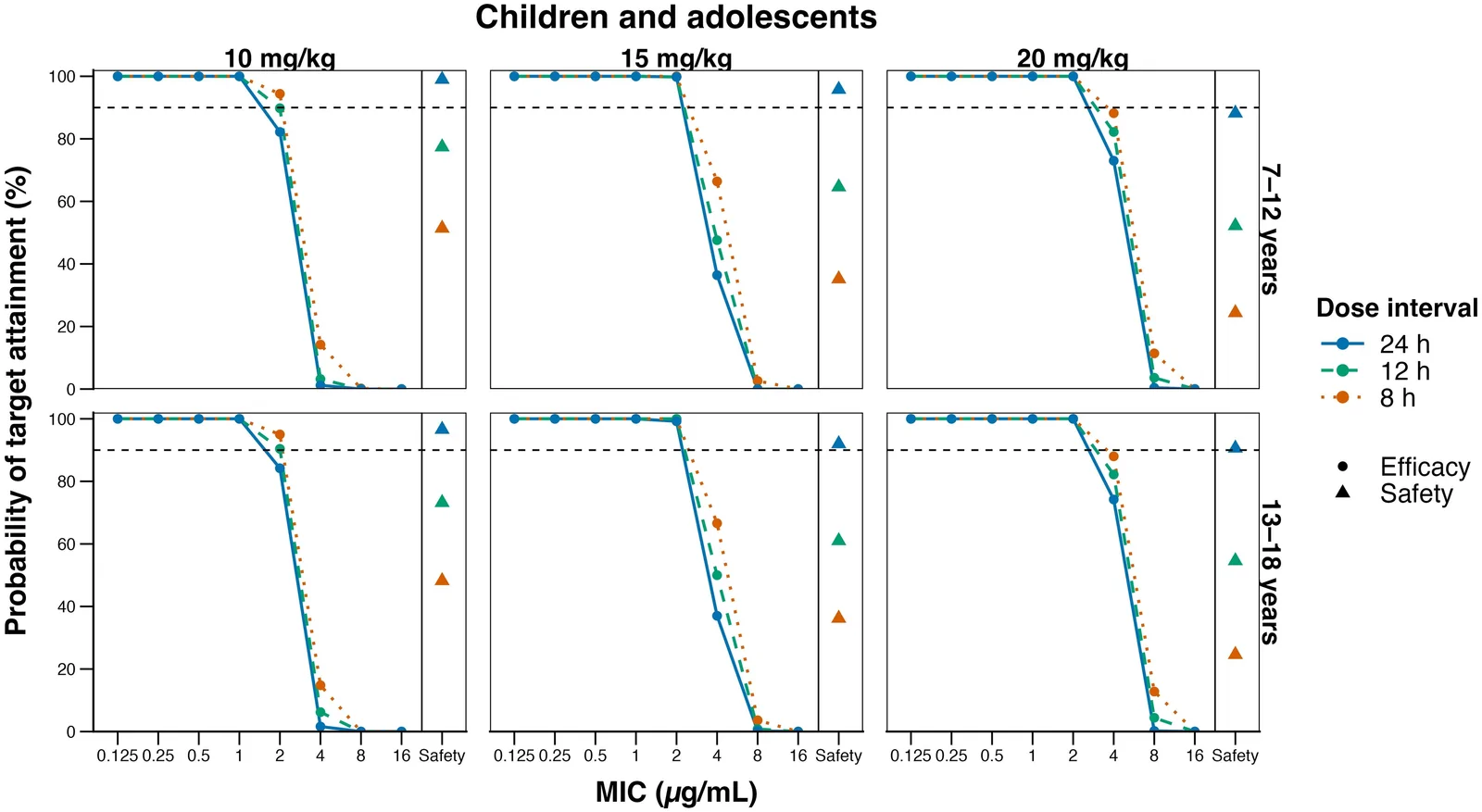
Probability of target attainment (PTA) for efficacy and safety across dosing regimens in in children and adolescents. Efficacy PTA was defined as the proportion of simulated individuals achieving a peak concentration-to-MIC ratio (*C*_max_/MIC) ≥ 10 across MIC values from 0.125 to 16 mg/L. Safety PTA was defined as the proportion achieving a trough concentration (*C*_min_) < 5 mg/L. The dashed horizontal line represents the 90% efficacy target threshold. Results are shown for dose levels of 10, 15 and 20 mg/kg and stratified by stratified by age group (7–12 years and 13–18 years). Lines represent different dosing intervals (every 24, 12 or 8 h).

### Discussion

An amikacin PopPK model was developed using neonatal data, and then externally evaluated for its predictive performance in an independent cohort of older paediatric patients without parameter re-estimation. The model developed using the neonatal data, incorporating mechanistic covariates previously shown to be predictive for other renally cleared antibiotics,^22,43^ demonstrated lower prediction bias and improved precision compared with previously published paediatric amikacin models. Following external evaluation, the model was refitted to the combined neonatal and older paediatric dataset to obtain final parameter estimates informed by PK data across the full paediatric age range. PTA simulations were also performed to inform potential dosing strategies.

Neonates were chosen as the starting point because they have the most immature renal function and the highest variability in amikacin clearance. The rapid maturation of glomerular filtration, postnatal renal development, and proportionally large extracellular fluid volumes make this population the most challenging from a PK standpoint. Building the model in neonates ensures that mechanistic maturation covariates (PMA-driven maturation, postnatal changes and age-adjusted creatinine) capture these early developmental changes. Once established, the model was projected onto older children, where renal maturation is more predictable and variability decreases. This approach allows a single PopPK model to predict amikacin PK from birth to 18 years, facilitating individualized dosing without requiring clinicians to estimate renal function separately for dose selection.

Although amikacin is often adequately described by a one-compartment model, a two-compartment structure provided a superior fit in this study. The primary covariate modelling focus was clearance, while V1, Q and V2 were scaled by body weight. The predominantly peak–trough sampling design provided limited information during the distribution phase. Although the covariance-based RSEs for Q and V2 were acceptable, bootstrap estimates for both parameters differed from the final estimates, and the high η-shrinkage warrants cautious interpretation of the distribution parameters. Despite its relatively simple PK profile, amikacin exhibits high IIV and a low therapeutic index. Previous studies have reported substantial variability that could not be fully explained by body weight and gestational age alone, highlighting the importance of explicitly modelling renal maturation.^12,17,44,45^

The wide variability in observed concentrations, especially in neonates receiving standard weight-based dosing, confirms that body weight alone is insufficient to predict exposure. These findings support the need for individualized dosing strategies beyond traditional weight-based approaches followed by TDM. *A priori* dose optimization based on patient-specific covariates may improve early target attainment and reduce the risk of toxicity, but Bayesian model–informed precision dosing should ideally be used to predict future doses.^46^ The model presented here would likely be a good basis for this given its predictive performance relative to the evaluated paediatric models (Figure 1).

The developed model integrated mechanistic covariates representing renal maturation and size-based scaling of clearance. Rhodin *et al*.’s renal function model was used to describe PMA-driven maturation and allometric scaling. SCr was evaluated as a covariate on clearance using a power function based on the ratio of the patient’s observed SCr to the mean age-adjusted SCr, calculated using the Ceriotti *et al*. formula. Postnatal clearance maturation and concomitant glycopeptide use were also included. This combined approach allowed both developmental and functional aspects of renal clearance to be represented, improving prediction accuracy across paediatric age groups.

The strong correlation between age-related covariates (PMA, PNA and weight) is expected in neonatal and paediatric PK datasets, reflecting developmental progression. However, such relationships may introduce multicollinearity issues if these variables are estimated simultaneously in a PK model.^47^ Scientifically justified inclusion of these covariates can be achieved by fixing covariate models, such as the fixed allometric and PMA functions in our model developed using the neonatal data.^47^

External evaluation in older paediatric patients demonstrated that the neonatal-based model retained predictive accuracy and precision when extrapolated to the older paediatric population. Slightly higher prediction errors in older children may reflect differences in age and dosing characteristics between the development and evaluation populations. Goodness-of-fit diagnostics and visual predictive checks supported overall model adequacy.

Comparison with previously published models showed lower RMSE and MdAPE and higher F20 and F30 for the developed model. Many published models rely primarily on body weight or simple age relationships without explicitly accounting for renal maturation. De Cock *et al*.^39^ developed their amikacin model in neonates; however, the wide interquartile range observed in the boxplot indicates substantial variability in individual predictions, despite a median IPE of 0.9 mg/L.

Models by Sherwin *et al*.^18^ and Yu *et al*.^19^ relied primarily on body weight without explicitly incorporating maturation or renal function. Illamola *et al.*^17^ was developed in neonates to compare creatinine clearance and postnatal age as predictors of amikacin clearance. Similarly, Sherwin *et al.*^40^ used current body weight and PMA as covariates without explicitly modelling renal function development. These covariate structures may not fully capture the developmental changes in renal function that occur across the paediatric age range, which may contribute to reduced predictive performance when the models are applied to a different paediatric population. This comparison reinforces the importance of incorporating mechanistically informed covariates when predicting drug behaviour across the whole paediatric age range.

The unavailability of some covariates may contribute to differences in predictive performance. The PK model by Botha *et al*.^16^ showed substantially larger prediction errors; this may stem from the use of birth weight as a covariate, which was not available in our dataset and had to be excluded. In any case, the relevance of birth weight in patients beyond the neonatal period is questionable. Similarly, models by Allegaert *et al*.^41,42^ included clinically relevant but unavailable covariates such as ventilation status and intrauterine growth retardation (SGA) score. These covariates were set to default values due to data limitations, which may have affected their predictive performance in our dataset.

Although AUC/MIC has been proposed as an alternative PK/PD index for amikacin, Cmax/MIC remains a clinically established efficacy target and has been widely used in previous amikacin PK/PD studies.^48–51^ Therefore, the present study evaluated PTA using the Cmax/MIC efficacy target. The PTA simulations indicated that higher doses (25–35 mg/kg) improved efficacy across most age groups but increased toxicity risk, particularly in early neonates. In contrast, low doses (5 mg/kg) consistently failed to achieve adequate target attainment. Comparable efficacy was observed across Q8h, Q12h and Q24h regimens; however, once-daily dosing at 10–15 mg/kg provided superior safety. These findings support Q24h dosing as a balanced strategy, especially in neonates and patients with impaired renal function.

PTA declined markedly at MIC values ≥8 mg/L, which have been associated with increased mortality in patients with Enterobacterales bacteraemia treated with amikacin.^52^ Given current European Committee on Antimicrobial Susceptability Testing (EUCAST) (≤16 mg/L for *Pseudomonas aeruginosa*; ≤8 mg/L for Enterobacterales) and Clinical and Laboratory Standards Institute (CLSI) (≤4 mg/L for Enterobacterales) breakpoints, these findings reinforce the importance of individualizing the initial dose that balances efficacy and toxicity, particularly in neonates and renally impaired patients.^52,53^

Although the model developed using neonatal data demonstrated acceptable predictive performance when externally evaluated in the older paediatric cohort, several limitations should be acknowledged. First, the final model, obtained by refitting to the combined neonatal and older paediatric dataset, has not undergone independent external evaluation and therefore requires further evaluation before clinical implementation. Second, the analysis was based on retrospectively collected data obtained during routine clinical care, and sampling times were therefore not prospectively standardized. Third, the study was conducted within a single centre, and additional evaluation in external institutions with different patient populations and clinical practices is warranted. Finally, although the study included patients from extremely preterm neonates through to adolescence and across diverse clinical conditions, not all clinical situations that may influence amikacin exposure were represented in the available dataset. Future work should therefore explore the integration of this model into MIPD frameworks to further evaluate its predictive performance in clinical practice.

### Conclusion

This study developed a maturation-informed PopPK model of amikacin in neonates that successfully predicted concentrations in an independent cohort of older paediatric patients. Subsequent refitting to the combined dataset provided a unified model with parameter estimates informed by patients across the full paediatric age range. The final model provides a framework for future model-informed dosing of amikacin in paediatric patients.

## Supplementary Material

dkag354_Supplementary_Data

## Supplementary data

Figure S1, Tables S1–S7 and Code S1 are available as Supplementary data at *JAC* Online.

## References

[1] Pacifici GM . Clinical pharmacokinetics of aminoglycosides in the neonate: a review. Eur J Clin Pharmacol 2009; 65: 419–27. 10.1007/s00228-008-0599-y 19104791

[2] Dowling PM, Prescott JF, Baptiste KE. Antimicrobial Therapy in Veterinary Medicine. John Wiley & Sons, 2025.

[3] Gilbert DN . Once-daily aminoglycoside therapy. Antimicrob Agents Chemother 1991; 35: 399–405. 10.1128/AAC.35.3.399 PMC245022 2039189

[4] McKellar QA, Sanchez Bruni SF, Jones DG. Pharmacokinetic/pharmacodynamic relationships of antimicrobial drugs used in veterinary medicine. J Vet Pharmacol Ther 2004; 27: 503–14. 10.1111/j.1365-2885.2004.00603.x 15601444

[5] Rotschafer JC, Andes DR, Rodvold KA. Antibiotic Pharmacodynamics. Humana Press, 2016.

[6] Fuursted K . Post-antibiotic effect of ciprofloxacin on Pseudomonas aeruginosa. Eur J Clin Microbiol 1987; 6: 271–4. 10.1007/BF02017611 3113938

[7] Rivetti S, Romano A, Mastrangelo S et al Aminoglycosides-related ototoxicity: mechanisms, risk factors, and prevention in pediatric patients. Pharmaceuticals (Basel) 2023; 16: 1353. 10.3390/ph16101353 PMC10610175 37895824

[8] Paediatric Formulary Committee . Amikacin BNF for children. http://www.medicinescomplete.com.

[9] Bio L, Frymoyer A. Guidelines for Aminoglycoside use. https://www.stanfordchildrens.org/content/dam/sch/content-public/pdf/antimicrobial-stewardship-program/aminoglycoside-guideline.pdf.

[10] Kato H, Hagihara M, Hirai J et al Evaluation of amikacin pharmacokinetics and pharmacodynamics for optimal initial dosing regimen. Drugs R D 2017; 17: 177–87. 10.1007/s40268-016-0165-5 PMC5318333 28063020

[11] Aréchiga-Alvarado NA, Medellín-Garibay SE, Milán-Segovia RDC et al Population pharmacokinetics of amikacin administered once daily in patients with different renal functions. Antimicrob Agents Chemother 2020; 64: e02178-19. 10.1128/AAC.02178-19 PMC7179627 32041715

[12] Bleyzac N, Varnier V, Labaune J et al Population pharmacokinetics of amikacin at birth and interindividual variability in renal maturation. Eur J Clin Pharmacol 2001; 57: 499–504. 10.1007/s002280100355 11699615

[13] Kearns GL, Abdel-Rahman SM, Alander SW et al Developmental pharmacology—drug disposition, action, and therapy in infants and children. N Engl J Med 2003; 349: 1157–67. 10.1056/NEJMra035092 13679531

[14] Guignard J-P, Drukker A. Why do newborn infants have a high plasma creatinine?. Pediatrics 1999; 103: e49. 10.1542/peds.103.4.e49. 10103341

[15] Touw DJ, Westerman EM, Sprij AJ. Therapeutic drug monitoring of aminoglycosides in neonates. Clin Pharmacokinet 2009; 48: 71–88. 10.2165/00003088-200948020-00001 19271781

[16] Botha J, Du Preez M, Miller R et al Determination of population pharmacokinetic parameters for amikacin in neonates using mixed-effect models. Eur J Clin Pharmacol 1998; 53: 337–41. 10.1007/s002280050389 9516033

[17] Illamola SM, Colom H, van Hasselt JC. Evaluating renal function and age as predictors of amikacin clearance in neonates: model-based analysis and optimal dosing strategies. Br J Clin Pharmacol 2016; 82: 793–805. 10.1111/bcp.13016 PMC5338126 27198625

[18] Sherwin CM, Wead S, Stockmann C et al Amikacin population pharmacokinetics among paediatric burn patients. Burns 2014; 40: 311–8. 10.1016/j.burns.2013.06.015 23876785

[19] Yu T, Stockmann C, Healy DP et al Determination of optimal amikacin dosing regimens for pediatric patients with burn wound sepsis. J Burn Care Res 2015; 36: e244–52. 10.1097/BCR.0000000000000159 25185930

[20] Germovsek E, Kent A, Metsvaht T et al Development and evaluation of a gentamicin pharmacokinetic model that facilitates opportunistic gentamicin therapeutic drug monitoring in neonates and infants. Antimicrob Agents Chemother 2016; 60: 4869–77. 10.1128/AAC.00577-16 PMC4958175 27270281

[21] Barker CIS, Kipper K, Lonsdale DO et al The neonatal and paediatric pharmacokinetics of antimicrobials study (NAPPA): investigating amoxicillin, benzylpenicillin, flucloxacillin and piperacillin pharmacokinetics from birth to adolescence. J Antimicrob Chemother 2023; 78: 2148–61. 10.1093/jac/dkad196 PMC10477139 37531085

[22] Lonsdale DO, Kipper K, Baker EH et al β-Lactam antimicrobial pharmacokinetics and target attainment in critically ill patients aged 1 day to 90 years: the ABDose study. J Antimicrob Chemother 2020; 75: 3625–34. 10.1093/jac/dkaa363 32989452

[23] Kane Z, Gastine S, Obiero C et al IV and oral fosfomycin pharmacokinetics in neonates with suspected clinical sepsis. J Antimicrob Chemother 2021; 76: 1855–64. 10.1093/jac/dkab083 PMC8212774 33855449

[24] Germovsek E, Lutsar I, Kipper K et al Plasma and CSF pharmacokinetics of meropenem in neonates and young infants: results from the NeoMero studies. J Antimicrob Chemother 2018; 73: 1908–16. 10.1093/jac/dky128 PMC6005047 29684147

[25] R Core Team . R: A Language and Environment for Statistical Computing. R Foundation for Statistical Computing, 2026.

[26] Guiastrennec B, Hooker AC, Ueckert S et al xpose: diagnostics for pharmacometric models. R package version 0.4.23. 2026. https://doi.org/10.32614/CRAN.package.xpose.

[27] Wickham H . ggplot2: Elegant Graphics for Data Analysis. Springer-Verlag, 2016.

[28] Beal SL . Ways to fit a PK model with some data below the quantification limit. J Pharmacokinet Pharmacodyn 2001; 28: 481–504. 10.1023/A:1012299115260 11768292

[29] Germovsek E, Barker CI, Sharland M et al Scaling clearance in paediatric pharmacokinetics: all models are wrong, which are useful? Br J Clin Pharmacol 2017; 83: 777–90. 10.1111/bcp.13160 PMC5346879 27767204

[30] Lonsdale DO, Baker EH, Kipper K et al Scaling beta-lactam antimicrobial pharmacokinetics from early life to old age. Br J Clin Pharmacol 2019; 85: 316–46. 10.1111/bcp.13756 PMC6339963 30176176

[31] Kleiber M . Body size and metabolism. Hilgardia 1932; 6: 315–53. 10.3733/hilg.v06n11p315

[32] Rhodin MM, Anderson BJ, Peters AM et al Human renal function maturation: a quantitative description using weight and postmenstrual age. Pediatr Nephrol 2009; 24: 67–76. 10.1007/s00467-008-0997-5 18846389

[33] Holford N, Heo Y-A, Anderson B. A pharmacokinetic standard for babies and adults. J Pharm Sci 2013; 102: 2941–52. 10.1002/jps.23574 23650116

[34] Ceriotti F, Boyd JC, Klein G et al Reference intervals for serum creatinine concentrations: assessment of available data for global application. Clin Chem 2008; 54: 559–66. 10.1373/clinchem.2007.099648 18202155

[35] Royle JA, Dorazio RM. Hierarchical Modeling and Inference in Ecology: the Analysis of Data from Populations, Metapopulations and Communities. Academic Press, 2008.

[36] Sheiner LB, Beal SL. Some suggestions for measuring predictive performance. J Pharmacokinet Biopharm 1981; 9: 503–12. 10.1007/BF01060893 7310648

[37] Pérez-Blanco JS, Sáez Fernández EM, Calvo MV et al Evaluation of current amikacin dosing recommendations and development of an interactive nomogram: the role of albumin. Pharmaceutics 2021; 13: 264. 10.3390/pharmaceutics13020264 PMC7919491 33672057

[38] Bland CM, Pai MP, Lodise TP. Reappraisal of contemporary pharmacokinetic and pharmacodynamic principles for informing aminoglycoside dosing. Pharmacotherapy 2018; 38: 1229–38. 10.1002/phar.2193 30403305

[39] De Cock RF, Allegaert K, Schreuder MF et al Maturation of the glomerular filtration rate in neonates, as reflected by amikacin clearance. Clin Pharmacokinet 2012; 51: 105–17. 10.2165/11595640-000000000-00000 22229883

[40] Sherwin CM, Svahn S, Van Der Linden A et al Individualised dosing of amikacin in neonates: a pharmacokinetic/pharmacodynamic analysis. Eur J Clin Pharmacol 2009; 65: 705–13. 10.1007/s00228-009-0637-4 19305985

[41] Allegaert K, Anderson BJ, Cossey V et al Limited predictability of amikacin clearance in extreme premature neonates at birth. Br J Clin Pharmacol 2006; 61: 39–48. 10.1111/j.1365-2125.2005.02530.x PMC1884978 16390350

[42] Allegaert K, Scheers I, Cossey V et al Covariates of amikacin clearance in neonates: the impact of postnatal age on predictability. Drug Metab Lett 2008; 2: 286–9. 10.2174/187231208786734157 19356107

[43] Colin PJ, Allegaert K, Thomson AH et al Vancomycin pharmacokinetics throughout life: results from a pooled population analysis and evaluation of current dosing recommendations. Clin Pharmacokinet 2019; 58: 767–80. 10.1007/s40262-018-0727-5 30656565

[44] Vučićević K, Rakonjac Z, Miljković B et al Pharmacokinetic variability of amikacin after once-daily and twice-daily dosing regimen in full-term neonates. J Pharmacol Sci 2014; 124: 138–43. 10.1254/jphs.13126FP 24441865

[45] Allegaert K, Anderson BJ. Interindividual variability of aminoglycoside pharmacokinetics in preterm neonates at birth. Eur J Clin Pharmacol 2006; 62: 1011–2. 10.1007/s00228-006-0204-1 17006675

[46] Wicha SG, Märtson AG, Nielsen EI et al From therapeutic drug monitoring to model-informed precision dosing for antibiotics. Clin Pharmacol Ther 2021; 109: 928–41. 10.1002/cpt.2202 33565627

[47] Bonate PL . The effect of collinearity on parameter estimates in nonlinear mixed effect models. Pharm Res 1999; 16: 709–17. 10.1023/A:1018828709196 10350015

[48] Ruiz J, Ramirez P, Company MJ et al Impact of amikacin pharmacokinetic/pharmacodynamic index on treatment response in critically ill patients. J Glob Antimicrob Resist 2018; 12: 90–5. 10.1016/j.jgar.2017.09.019 29017888

[49] Kato H, Parker SL, Roberts JA et al Population pharmacokinetics analysis of amikacin initial dosing regimen in elderly patients. Antibiotics (Basel) 2021; 10: 100. 10.3390/antibiotics10020100 PMC7909551 33498481

[50] Sturkenboom MG, Simbar N, Akkerman OW et al Amikacin dosing for MDR tuberculosis: a systematic review to establish or revise the current recommended dose for tuberculosis treatment. Clin Infect Dis 2018; 67: S303–7. 10.1093/cid/ciy613 30496466

[51] Boidin C, Bourguignon L, Cohen S et al Amikacin initial dose in critically ill patients: a nonparametric approach to optimize *a priori* pharmacokinetic/pharmacodynamic target attainments in individual patients. Antimicrob Agents Chemother 2019; 63: e00993-19. 10.1128/AAC.00993-19 PMC6811403 31481443

[52] Zohar I, Ben David D, Schwartz O et al Amikacin treatment in patients with enterobacterales bacteraemia: impact of MIC on mortality. J Antimicrob Chemother 2024; 79: 3204–9. 10.1093/jac/dkae343 39331516

[53] European Committee on Antimicrobial Susceptibility Testing . Breakpoint tables for interpretation of MICs and zone diameters. https://www.eucast.org/fileadmin/eucast/pdf/breakpoints/v_16.0_Breakpoint_Tables.pdf.

