# Supplementary material for: A unified population pharmacokinetic model of amikacin in paediatric patients from birth to 18 years for model-informed precision dosing
Source: J Antimicrob Chemother. 2026 Oct 22;81(11):dkag354. doi: 10.1093/jac/dkag354 (PMC13688210; doi:10.1093/jac/dkag354)
Supplement: Supplementary file 1 [file dkag354_supplementary_data.docx]

**Supplementary**

Figure S1 Goodness-of-fit plots for the model developed using neonatal data. (A) Observed concentrations (DV) versus population predictions (PRED); (B) DV versus individual predictions (IPRED); (C) normalised prediction distribution errors (NPDE) versus time after dose (TAD); (D) NPDE versus PRED; (E) conditional weighted residuals (CWRES) versus PRED; and (F) absolute individual weighted residuals (|IWRES|) versus IPRED. The red line represents a smoothed curve that illustrates the general pattern of the data. In panels A and B, the black diagonal line represents the line of identity ($y=x$). In panels C–E, the solid horizontal line indicates zero and the dashed horizontal lines indicate ±2.

| **Structure** | | | | **Model** | **OFV** | **ΔOFV** | **Compared to model** | **Model selected** |
| --- | --- | --- | --- | --- | --- | --- | --- | --- |
| **Type** | **IIV** | **IIV covariance** | **RUV** |  |  |  |  |  |
| One-Compartment | CL | – | add | Run 1 | 2782.276 | _ | – | – |
| One-Compartment | CL | – | prop | Run 2 | 2512.075 | -270.201 | Run1 | Run2 |
| One-Compartment | CL | – | add+prop | Run 3 | 2453.755 | -58.32 | Run2 | Run3 |
| One-Compartment | CL +V1 | Diagonal | add | Run 4 | 2803.518 | 349.763 | Run3 | Run3 |
| One-Compartment | CL +V1 | Block | add | Run 5 | 2785.381 | 331.626 | Run3 | Run3 |
| One-Compartment | CL +V1 | Block | prop | Run 6 | 2769.28 | 315.525 | Run3 | Run3 |
| One-Compartment | CL +V1 | Block | add+prop | Run 7 | 2412.964 | -40.791 | Run3 | Run7 |
| Two-Compartment | CL | Diagonal | add | Run 8 | 2769.948 | 356.984 | Run7 | Run7 |
| Two-Compartment | CL | Diagonal | add+prop | Run 9 | 2489.515 | 76.551 | Run7 | Run7 |
| Two-Compartment | CL+V1 | Block | add | Run 10 | 2775.464 | 362.5 | Run7 | Run7 |
| Two-Compartment | CL+V1 | Block | add+prop | Run 11 | 2373.369 | -39.595 | Run7 | Run11 |
| Two-Compartment | CL+V1+V2+Q | Block | add+prop | **Run12** | **2341.434** | **-31.935** | Run11 | **Run12** |
| Two-Compartment | CL+V1+V2 | Block | add+prop | Run 13 | 2395.633 | 54.199 | Run12 | Run12 |
| Two-Compartment | CL+V1+Q | Block | add+prop | Run 14 | 2357.856 | 16.422 | Run12 | Run12 |
| **Type:** PK Compartment Model. **IIV:** Inter-individual variability. **IIV covariance:** Diagonal indicates that IIV terms were estimated without covariance between random effects; block indicates that covariance between random effects was estimated. **RUV:** Residual Unexplained Variability. **add:** additive error model, **prop:** proportional error model, **add+prop:** combined error model. **ΔOFV:** change in objective function value. Parameter abbreviations: **CL** – clearance (L/h); **V1** – central volume of distribution (L); **Q** – intercompartmental clearance (L/h); **V2** – peripheral volume of distribution (L). All models were estimated using FOCEI (First-Order Conditional Estimation with Interaction). | | | | | | | | |

Table S1 Summary of key structural, IIV, and residual error model-development steps

Table S2 Summary of the key steps in covariate model development and selection

| **Hypothesis** | **Model** | **OFV** | **ΔOFV** | **Compared to model** | **Model selected** |
| --- | --- | --- | --- | --- | --- |
| **Final base model** | Run 12 | 2133.75 | - | - | - |
|  |  |  |  |  |  |
| **+ WT on CL + WT on V1 + WT on Q  + WT on V2** | Run 15 | 2215.554 | -125.88 | Run 12 | Run 15 |
| $TVCL= \left[ CL \times\left( \frac{{WT}_{i}}{70} \right)^{\beta_{WT}} \right]$ |  |  |  |  |  |
| $TVV1= \left[ V1 \times\left( \frac{{WT}_{i}}{70} \right)^{\beta_{WT}} \right]$ |  |  |  |  |  |
| $TVQ = \left[ Q \times\left( \frac{{WT}_{i}}{70} \right)^{\beta_{WT}} \right]$ |  |  |  |  |  |
| $TVV2= \left[ V2 \times\left( \frac{{WT}_{i}}{70} \right)^{\beta_{WT}} \right]$ |  |  |  |  |  |
| **+ WT on CL + PMA on CL + WT on V1 + WT on Q  + WT on V2** | Run 16 | 2204.304 | -11.25 | Run 15 | Run 16 |
| $TVCL= \left[ CL \times\left( \frac{{WT}_{i}}{70} \right)^{\beta_{WT}}\times\left( \frac{{{PMA}_{i}}^{3.4}}{{47.7}^{3.4}+{{PMA}_{i}}^{3.4}} \right) \right]$ |  |  |  |  |  |
| $TVV1= \left[ V1 \times\left( \frac{{WT}_{i}}{70} \right)^{\beta_{WT}} \right]$ |  |  |  |  |  |
| $TVQ = \left[ Q \times\left( \frac{{WT}_{i}}{70} \right)^{\beta_{WT}} \right]$ |  |  |  |  |  |
| $TVV2= \left[ V2 \times\left( \frac{{WT}_{i}}{70} \right)^{\beta_{WT}} \right]$ |  |  |  |  |  |
| **+ WT on CL + PMA on CL + Scr on CL + WT on V1 + WT on Q  + WT on V2** | Run 17 | 2152.33 | -63.224 | Run 16 | Run 17 |
| $TVCL= \left[ CL \times\left( \frac{{WT}_{i}}{70} \right)^{\beta_{WT}}\times\left( \frac{{{PMA}_{i}}^{3.4}}{{47.7}^{3.4}+{{PMA}_{i}}^{3.4}} \right) \times\left( \frac{{Scr}_{i}}{TSCR} \right)^{\beta_{Scr}} \right]$ |  |  |  |  |  |
| $TVV1= \left[ V1 \times\left( \frac{{WT}_{i}}{70} \right)^{\beta_{WT}} \right]$ |  |  |  |  |  |
| $TVQ = \left[ Q \times\left( \frac{{WT}_{i}}{70} \right)^{\beta_{WT}} \right]$ |  |  |  |  |  |
| $TVV2= \left[ V2 \times\left( \frac{{WT}_{i}}{70} \right)^{\beta_{WT}} \right]$ |  |  |  |  |  |
|  |  |  |  |  |  |
| **+ WT on CL + PMA on CL + Scr on CL + PNA on CL + WT on V1 + WT on Q  + WT on V2** | Run 18 | 2132.044 | -20.286 | Run 17 | Run 18 |
| $TVCL= \left[ \begin{aligned} CL \times\left( \frac{{WT}_{i}}{70} \right)^{\beta_{WT}} \times\left( \frac{{{PMA}_{i}}^{3.4}}{{47.7}^{3.4}+{{PMA}_{i}}^{3.4}} \right) \times\left( \frac{{Scr}_{i}}{TSCR} \right)^{\beta_{Scr}} \\ \times\left[ hM+(1-hM)\times(1-\exp\left( -PNA\times\mathrm{hN} \right)) \right] \end{aligned} \right]$ |  |  |  |  |  |
| $TVV1= \left[ V1 \times\left( \frac{{WT}_{i}}{70} \right)^{\beta_{WT}} \right]$ |  |  |  |  |  |
| $TVQ = \left[ Q \times\left( \frac{{WT}_{i}}{70} \right)^{\beta_{WT}} \right]$ |  |  |  |  |  |
| $TVV2= \left[ V2 \times\left( \frac{{WT}_{i}}{70} \right)^{\beta_{WT}} \right]$ |  |  |  |  |  |
|  |  |  |  |  |  |
| **+ WT on CL + PMA on CL + Scr on CL + PNA on CL + glycopeptides on CL + WT on V1 + WT on Q  + WT on V2** | **Run 19** | 2087.954 | -44.09 | **Run 18** | **Run19**  **(Final neonatal model)** |
| $TVCL= \left[ \begin{aligned} CL \times\left( \frac{{WT}_{i}}{70} \right)^{\beta_{WT}} \times\left( \frac{{{PMA}_{i}}^{3.4}}{{47.7}^{3.4}+{{PMA}_{i}}^{3.4}} \right) \times\left( \frac{{Scr}_{i}}{TSCR} \right)^{\beta_{Scr}} \\ \times\left[ hM+(1-hM)\times(1-\exp\left( -PNA\times\mathrm{hN} \right)) \right] \times\left( \mathbf{glycopeptides} \right)^{\beta_{\mathrm{glycopeptide}}} \end{aligned} \right]$ |  |  |  |  |  |
| $TVV1= \left[ V1 \times\left( \frac{{WT}_{i}}{70} \right)^{\beta_{WT}} \right]$ |  |  |  |  |  |
| $TVQ = \left[ Q \times\left( \frac{{WT}_{i}}{70} \right)^{\beta_{WT}} \right]$ |  |  |  |  |  |
| $TVV2= \left[ V2 \times\left( \frac{{WT}_{i}}{70} \right)^{\beta_{WT}} \right]$ |  |  |  |  |  |
|  |  |  |  |  |  |
| Table S2 presents the key retained steps of covariate model development following forward inclusion and backward elimination; not all models tested during the covariate selection procedure are shown.  Parameter abbreviations: **CL** – clearance (L/h); **V1** – central volume of distribution (L); **Q** – intercompartmental clearance (L/h); **V2** – peripheral volume of distribution (L); **ηBSV** – between-subject variability. Covariate abbreviations: **WT** – body weight (scaled to 70 kg); **WTi –** current body weight ; **PMA** – postmenstrual age function; **Scr** – age-adjusted mean serum creatinine; **PNA** – postnatal age function; **glycopeptides** – concomitant use of glycopeptides. **βWT** – fixed allometric exponent for body weight scaling (0.75 for CL and Q; 1.0 for V1 and V2). | | | | | |

| Table S3 Accuracy and precision of the final neonatal model and model evaluation | | |  |
| --- | --- | --- | --- |
|  | **Final neonatal model** | **Model evaluation** | |
| **PRED vs OBS** |  |  | |
| Mean prediction error (mg/L) | -0.85 | -3.89 | |
| Root mean squared error (mg/L) | 7.07 | 8.17 | |
| **IPRED vs OBS** |  |  | |
| Mean prediction error (mg/L) | -0.19 | -0.80 | |
| Root mean squared error (mg/L) | 5.32 | 3.15 | |

| Table S4 Summary of Published Amikacin Pharmacokinetic Models in Children | | | | | | | | | |
| --- | --- | --- | --- | --- | --- | --- | --- | --- | --- |
| **paper** | **Population** | **Sample Size** | **Model** | **Parameter** | | **Description (unit)** | **Estimate** | **Covariate Effect** | **Model equation** |
| **De Cock et al . (2012) ^1^** | Neonates | 874 | Two- comp | Θ1 | CL |  | 0.0493 | bBW, PNA, ibuprofen | CL=[(Θ1) * (bBW/median bBW)^(Θ5) * (1+ Θ6 *(PNA/median PNA))×(Θ7)]* exp(ηBSV-CL)  V1= [(Θ2) * (cBW/median cBW)^(Θ8)]* exp(ηBSV-V) |
|  |  |  |  | Θ2 | V1 |  | 0.833 | cBW |  |
|  |  |  |  | Θ3 | Q |  | 0.415 | - |  |
|  |  |  |  | Θ4 | V2 |  | 0.833 | - |  |
|  |  |  |  | Θ5 | CL~bBW | birth Weight (g) | 1.34 | - |  |
|  |  |  |  | Θ6 | CL~PNA | postnatal age (d) | 0.213 | - |  |
|  |  |  |  | Θ7 | CL~IBU | ibuprofen [is a binary variable (1 if not administered, 0.838 if administered)] | 0.838 | - |  |
|  |  |  |  | Θ8 | V~cBW | current weight (g) | 0.919 | - |  |
|  |  |  |  |  | ηBSV-CL |  | 0.0899 | - |  |
|  |  |  |  |  | s2 | proportional error | 0.0614 | - |  |
|  |  |  |  |  | s2 | additive error | 0.267 | - |  |
| **Sherwin et al . (2009) ^2^** | Neonates | 80 | One- comp | Θ1 | CL |  | 0.23 | cBW, PMA | CL=[(Θ1) * (cBW/2)^(Θ3) * (PMA/40)^(Θ4)]* exp(ηBSV-CL) |
|  |  |  |  | Θ2 | V |  | 0.957 | cBW |  |
|  |  |  |  | Θ3 | CL~cBW | current Weight | 0.691 | - | V= [(Θ2) * (cBW/2)^(Θ5)]* exp(ηBSV-V) |
|  |  |  |  | Θ4 | CL~PMA | postmenstrual Age | 3.23 | - |  |
|  |  |  |  | Θ5 | V~cBW | current Weight | 0.89 | - |  |
|  |  |  |  |  | ηBSV-CL |  | 0.002 | - |  |
|  |  |  |  |  | ηBSV-V |  | 0.00002 | - |  |
|  |  |  |  |  | s2 | proportional error | 0.00144 | - |  |
|  |  |  |  |  | s2 | additive error | 2.42 | - |  |
| **Allegaert et al . (2006) ^3^** | Neonates | 205 | One- comp | Θ1 | CL | standardised to a 70 kg person | 0.486 | cBW, postconceptional age (PCA), scaling factor for premature neonates given a NSAID (once or twice per day) | CL= [(Θ1) * (cBW/70)^(Θ3)] * [exp(Θ4*(PCA-24)*Θ5)] * exp(ηBSV-CL)  V1= [(Θ2) * (cBW/70)]* exp(ηBSV-V) |
|  |  |  |  | Θ2 | V | standardised to a 70 kg person | 40.2 | cBW |  |
|  |  |  |  | Θ3 | CL~cBW | current Weight | 0.75 | - |  |
|  |  |  |  | Θ4 | CL~SLPCL | is the factor relating postconceptional age (PCA) to developmental changes in CL at 24 weeks | 0.11 | - |  |
|  |  |  |  | Θ5 | CL~NSAID | NSAID use | 0.788 | - |  |
|  |  |  |  |  | ηBSV-CL |  | 0.11 | - |  |
|  |  |  |  |  | ηBSV-V |  | 0.2 | - |  |
|  |  |  |  |  | s2 | additive error | 1.59 | - |  |
| **Allegaert et al. (2008) ^4^** | Neonates | 715 | One- comp | Θ1 | CL | standardised to a 70 kg person | 1.49 | cBW, PMA, SGA, RF, inotropes, artificiel ventilation | CL=(Θ1) * [(cBW/70)^(Θ3)] * [1+Θ4 *(PMA-40] * [Θ5 *VENT] * [Θ6*Ino] * [Θ7*SGA] *RF* exp(ηBSV-CL) |
|  |  |  |  | Θ2 | V | standardised to a 70 kg person | 31.7 | cBW, PNA, inotropes, artificial ventilation |  |
|  |  |  |  | Θ3 | CL~cBW | current Weight | 0.75 | - | V=(Θ2) * (cBW/70) * [1+Θ8 *PNA]* [Θ9* Ino] *[Θ10*VENT]* exp(ηBSV-V) |
|  |  |  |  | Θ4 | CL~ SLPCL | slope for PMA effect (1/week) | 0.032 | - |  |
|  |  |  |  | Θ5 | CL~Fvent | scaling factor for ventilation | 0.977 | - |  |
|  |  |  |  | Θ6 | CL~Finot | scaling factor for inotropes | 0.945 | - |  |
|  |  |  |  | Θ7 | CL~Fiugr | scaling factor for growth restriction | 0.872 | - |  |
|  |  |  |  | Θ8 | V~SLPV | slope for PNA effect (1/day) | 0.005 | - |  |
|  |  |  |  | Θ9 | V~Finot | Scaling factor for inotropes | 1.09 | - |  |
|  |  |  |  | Θ10 | V~Fvent | scaling factor for ventilation | 1.08 | - |  |
|  |  |  |  | Θ11 | CLcr~Kage | scaling constant for age | 0.00344 | - |  |
|  |  |  |  | Θ12 | CLcr~sRF | standardised to a 70 kg person | 6 | - | CPR=516*exp[Θ11 *( 52−40/PMA−30 )] Clcr= CPR/Scr RF = Clcr/Θ12 |
|  |  |  |  |  | ηBSV-CL |  | 0.013 | - |  |
|  |  |  |  |  | ηBSV-V |  | 0.0064 | - |  |
|  |  |  |  |  | s2 | proportional error | 0.18 | - |  |
|  |  |  |  |  | s2 | additive error | 0.644 | - |  |
| **Botha et al. (1998) ^5^** | Neonates | 53 | One- comp | Θ1 | CL |  | 0.031 | SEX, bBW | CL=Θ1×bBW^Θ3 *Θ4 *exp(ηBSV-CL)  V=Θ2×bBW^Θ5 *exp(ηBSV-V) |
|  |  |  |  | Θ2 | V |  | 0.316 | bBW |  |
|  |  |  |  | Θ3 | CL~bBW | exponent for birth weight | 1.45 | - |  |
|  |  |  |  | Θ4 | CL~SEX | gender multiplier (where =1.28 for girls and 1.0 for boys) | 1.28 | - |  |
|  |  |  |  | Θ5 | V~bBW | exponent for birth weight | 1.44 | - |  |
|  |  |  |  |  | ηBSV-CL |  | 0.032 | - |  |
|  |  |  |  |  | ηBSV-V |  | 0.017 | - |  |
|  |  |  |  |  | s2 | proportional error | 0.085 | - |  |
| **Illamola et al. (2016) ^6^** | Neonates | 149 | Two- comp | Θ1 | CL |  | 0.093 | cBW, Clcr | CL=[(Θ1) * (cBW/median cBW)^(Θ5) * ((Clcr/median Clcr)^(Θ6)]* exp(ηBSV-CL)  V =[(Θ2) * (cBW/median cBW)^(Θ7)]* exp(ηBSV-V1)  Q =[(Θ3) * (cBW/median cBW)^(Θ8)]* exp(ηBSV-Q)  CLcr= [K*Length (cm)]/[S creatinine (mg/dL)] where: K = 0.33 for premature neonates (GA<37) K = 0.45 for term infants (GA≥ 37) |
|  |  |  |  | Θ2 | V1 |  | 0.641 | cBW |  |
|  |  |  |  | Θ3 | Q |  | 0.042 | cBW |  |
|  |  |  |  | Θ4 | V2 |  | 0.478 | - |  |
|  |  |  |  | Θ5 | CL~cBW | current Weight (kg) | 0.799 | - |  |
|  |  |  |  | Θ6 | CL~CLcr | factor for creatinine clearance using Schwartz formula | 0.659 | - |  |
|  |  |  |  | Θ7 | V~cBW | current weight (kg) | 1.04 | - |  |
|  |  |  |  | Θ8 | Q~cBW | current weight (kg) | 0.909 | - |  |
|  |  |  |  |  | ηBSV-CL |  | 0.123 | - |  |
|  |  |  |  |  | ηBSV-V1 |  | 0.045 | - |  |
|  |  |  |  |  | ηBSV-Q |  | 0.255 | - |  |
|  |  |  |  |  | s2 | proportional error | 0.507 | - |  |
|  |  |  |  |  | s2 | additive error | 0.283 | - |  |
| **Yu et al. (2015) ^7^** | Children  (age 0.08–18 years) | 70 | one- comp | Θ1 | CL |  | 7.22 | cBW | CL=(Θ1) * (cBW/70) ^Θ3 * exp(ηBSV-CL)  V=(Θ2) * (cBW/70) * exp(ηBSV-V) |
|  |  |  |  | Θ2 | V |  | 22.7 | cBW |  |
|  |  |  |  | Θ3 | CL~cBW | current weight (kg) | 0.75 | - |  |
|  |  |  |  |  | ηBSV-CL |  | 0.06 | - |  |
|  |  |  |  |  | ηBSV-V1 |  | 0.01 (fix) | - |  |
|  |  |  |  |  | s2 | proportional error | 0.036 | - |  |
|  |  |  |  |  | s2 | additive error | 3.23 | - |  |
|  | Children  (age 0.08–18 years) | 32 | one- comp | Θ1 | CL |  | 5.36 | cBW | CL=(Θ1) * (cBW/70) ^Θ3 * exp(ηBSV-CL)  V=(Θ2) * (cBW/70) * exp(ηBSV-V) |
|  |  |  |  | Θ2 | V |  | 18.7 | cBW |  |
|  |  |  |  | Θ3 | CL~cBW | current weight (kg) | 0.75 | - |  |
|  |  |  |  |  | ηBSV-CL |  | 0.302 | - |  |
|  |  |  |  |  | ηBSV-V1 |  | 0.01 (fix) | - |  |
|  |  |  |  |  | s2 | proportional error | 0.0379 | - |  |
|  |  |  |  |  | s2 | additive error | 0.249 | - |  |
| **Sherwin et al. (2014) ^8^** | Infants/ children/ adolescents (burns) ( age 0.6–17 years) | 70 | two- comp | Θ1 | CL |  | 5.98 | cBW | CL=(Θ1) * (cBW/70) ^Θ5 * exp(ηBSV-CL)  V=(Θ2) * (cBW/70) * exp(ηBSV-V) |
|  |  |  |  | Θ2 | V1 |  | 16.7 | cBW |  |
|  |  |  |  | Θ3 | Q |  | 3.38 | - |  |
|  |  |  |  | Θ4 | V2 |  | 40.1 | - |  |
|  |  |  |  | Θ5 | CL~cBW | current Weight (kg) | 0.75 | - |  |
|  |  |  |  |  | ηBSV-CL |  | 0.0874 | - |  |
|  |  |  |  |  | ηBSV-V1 |  | 0.06 | - |  |
|  |  |  |  |  | ηBSV-Q |  | 0.01 (fix) | - |  |
|  |  |  |  |  | s2 | proportional error | 0.0385 | - |  |
|  |  |  |  |  | s2 | additive error | 1.81 | - |  |
| Summary of published population pharmacokinetic (PopPK) models of amikacin in paediatric populations. Each study includes information on study reference, population characteristics, sample size, model structure, estimated pharmacokinetic parameters, covariate effects, and final model equations. Parameter abbreviations: CL – Clearance (L/h); V1 – Volume of distribution in the central compartment (L); Q – Inter-compartmental clearance (L/h); V2 – Volume of distribution in the peripheral compartment (L); ηBSV between-subject variability. | | | | | | | | | |

| Table S5 Comparison of parameter estimates for the model developed using neonatal data and the final model,  with bootstrap evaluation of the final model. | | | | | | | | | |
| --- | --- | --- | --- | --- | --- | --- | --- | --- | --- |
|  | **Model developed using neonatal data** | | | **Final model** | | | **Bootstrap of the final model** | | |
|  | Estimate | %RSE | η-shrinkage (%) | Estimate | %RSE | η-shrinkage (%) | 2.5th percentile | 97.5th percentile | |
| Parameters |  |  |  |  |  |  |  |  | |
| CL (L/h/70 kg) | 5.63 | 0.52 | — | 5.63 | 0.28 | — | 4.70 | 5.72 | |
| V1 (L/70 kg) | 29.35 | 0.39 | — | 29.34 | 0.09 | — | 27.93 | 31.51 | |
| Q (L/h/70 kg) | 1.07 | 49.64 | — | 1.07 | 19.81 | — | 0.51 | 0.78 | |
| V2 (L/70 kg) | 30.26 | 0.75 | — | 30.26 | 0.51 | — | 44.1 | 85.33 | |
| β Scr (CL) | -0.73 | 1.67 | — | -0.73 | 0.63 | — | -1.04 | -0.75 | |
| β glycopeptides (CL) | -0.21 | 14.4 | — | -0.21 | 2.36 | — | -0.095 | -0.036 | |
| hM (CL) | 0.44 | 2.78 | — | 0.44 | 1.38 | — | 0.016 | 0.276 | |
| hN (CL) | 0.28 | 3.02 | — | 0.28 | 1.12 | — | 0.227 | 0.619 | |
| Between-subject variability |  |  |  |  |  |  |  |  | |
| BSV-CL (CV%) | 49.66 | — | 20.52 | 49.93 | — | 11.29 | — | — | |
| BSV-V1 (CV%) | 22.64 | — | 52.66 | 22.80 | — | 51.44 | — | — | |
| BSV-Q (CV%) | 91.33 | — | 49.97 | 91.40 | — | 41.36 | — | — | |
| BSV-V2 (CV%) | 32.69 | — | 67.6 | 32.70 | — | 72.45 | — | — | |
| Residual variability |  |  |  |  |  |  |  |  | |
| Proportional error (%) | 25 | 2.21 | — | 25 | 1.82 | — | 29 | 40 | |
| Additive error (mg/L) | 1.02 | 3.63 | — | 1.02 | 3.15 | — | 0.69 | 0.94 | |
| Abbreviations: CL, clearance; V1, central volume of distribution; Q, intercompartmental clearance; V2, peripheral volume of distribution; BSV, between-subject variability; RSE, relative standard error; SCr, serum creatinine. BSV is expressed as coefficient of variation (CV%). Bootstrap results are presented as the 2.5th and 97.5th percentiles from 1,000 non-parametric bootstrap replicates. CL, V1, Q and V2 bootstrap estimates are presented on the back-transformed scale. | | | | | | | | |  |

| Table S6 Predictive performance of the model developed using neonatal data and previously published paediatric amikacin population pharmacokinetic models in the older paediatric dataset | | | | | |
| --- | --- | --- | --- | --- | --- |
| **Model** | **Median PE (IPE), mg/L** | **RMSE (individual RMSE) , mg/L** | **MdAPE  (MdAIPE), %** | **F20 (IF20), %** | **F30  (IF30), %** |
| Current study | -0.03  (*-0.1*) | 6.2  (2.46) | 42.55  (*18.29*) | 22.22  (*53.27*) | 33.99  (*66.34*) |
| Allegaert (2008) | 0.77  (*0.61*) | 11.29  (7.89) | 86.91  (*67.77)* | 2.29  (*10.46*) | 4.25  (*17.97*) |
| Sherwin (2009) | 1  (*1*) | 11.99  (11.99) | 100  (*100*) | 1.96  (*1.96*) | 3.27  (*3.59*) |
| Illamola (2016) | -5.87  (*-0.57*) | 9.55  (7.23) | 93.27  (*23.36*) | 14.38  (*40.85*) | 22.55  (*82.42*) |
| Allegaert (2006) | 1  (*1*) | 12.83  (12.83) | 100  (*100*) | 0  (*0*) | 0  (*0*) |
| Botha (1998) | -95.75  (-77.83) | 463.53  (411.28) | 2655.55  (*2380.2*) | 0.98  (*5.23*) | 1.31  (*6.54*) |
| Yu (2015)B | 0.8  (*0.01*) | 6.24  (3.86) | 99.29  (*24.83*) | 11.76  (*46.41*) | 16.34  (*53.27*) |
| Yu (2015)A | 0.9  (*0.8*) | 7.16  (5.49) | 99.75  (*99.2*) | 6.86  (*16.67*) | 11.44  (*22.88*) |
| De Cock (2012) | 1  (*0.9*) | 12.46  (11.57) | 100  (*100*) | 0  (*3.92*) | 0  (*6.86*) |
| Sherwin (2014) | -0.8  (*-0.73*) | 8.98  (5.19) | 66.84  (*38.48*) | 9.15  (*25.16*) | 17.97  (*37.91*) |
| Values are presented for population predictions, with corresponding individual prediction metrics in parentheses. PE, prediction error; IPE, individual prediction error; RMSE, root mean squared error; MdAPE, median absolute percentage prediction error; MdAIPE, median absolute individual percentage prediction error; F20 and F30, percentages of predictions within ±20% and ±30% of the observed concentration, respectively; IF20 and IF30, corresponding percentages for individual predictions. PE was calculated as observed concentration − predicted concentration. Percentage-based metrics were calculated using quantifiable observations. | | | | | |

| Table S7 Comparison of Predicted Efficacy and Toxicity Across Different Dose Frequencies and Age Groups | | | | | | | | | | |
| --- | --- | --- | --- | --- | --- | --- | --- | --- | --- | --- |
| **Age group** | | **Dose interval  (hr)** | **MIC (mg/L)** | **PTA% Efficacy  at dose (mg/kg)** | | | **PTA% Safety  at dose (mg/kg)** | | | |
|  |  |  |  | **10** | **15** | **20** | **10** | **15** | **20** | |
| **Neo (PNA≤7 days)** | | **24** | 2 | 90.2 | 99.8 | 100 | 66 | 55.6 | 38.8 | |
|  |  |  | 4 | 5.8 | 57 | 87.8 |  |  |  | |
|  |  | **12** | 2 | 99 | 100 | 100 | 23 | 13 | 10.2 | |
|  |  |  | 4 | 29.4 | 83 | 95.2 |  |  |  | |
|  |  | **8** | 2 | 99.4 | 100 | 100 | 9 | 4.4 | 2 | |
|  |  |  | 4 | 52.4 | 91.6 | 98.2 |  |  |  | |
| **Neo (PNA>7 days)** | | **24** | 2 | 89.4 | 100 | 100 | 81.6 | 70.8 | 61.2 | |
|  |  |  | 4 | 4.4 | 47.8 | 80 |  |  |  | |
|  |  | **12** | 2 | 93.6 | 100 | 100 | 48.6 | 36.2 | 28.6 | |
|  |  |  | 4 | 15.8 | 66.8 | 87.2 |  |  |  | |
|  |  | **8** | 2 | 97.2 | 100 | 100 | 26.4 | 18.6 | 13.6 | |
|  |  |  | 4 | 32 | 78.8 | 92.6 |  |  |  | |
| **1 month –  2 years** | | **24** | 2 | 84 | 100 | 100 | 88 | 86 | 79.4 | |
|  |  |  | 4 | 2.8 | 37.8 | 70.4 |  |  |  | |
|  |  | **12** | 2 | 89.2 | 99.8 | 99.8 | 66.8 | 54.6 | 43.8 | |
|  |  |  | 4 | 11.2 | 54.6 | 81 |  |  |  | |
|  |  | **8** | 2 | 93 | 100 | 100 | 42.8 | 34 | 28.4 | |
|  |  |  | 4 | 20.6 | 69.4 | 88 |  |  |  | |
| **3 – 6 years** | | **24** | 2 | 80.8 | 100 | 100 | 98.2 | 94.8 | 90.4 | |
|  |  |  | 4 | 0.6 | 37.6 | 69.6 |  |  |  | |
|  |  | **12** | 2 | 89.2 | 100 | 100 | 79.6 | 65 | 57.2 | |
|  |  |  | 4 | 5.8 | 51.6 | 78.6 |  |  |  | |
|  |  | **8** | 2 | 95.2 | 100 | 100 | 52.4 | 39.6 | 26 | |
|  |  |  | 4 | 15.4 | 64.6 | 87.6 |  |  |  | |
| **7 – 12 years** | | **24** | 2 | 82.2 | 99.8 | 100 | 99 | 95.8 | 88.2 | |
|  |  |  | 4 | 1.2 | 36.4 | 73 |  |  |  | |
|  |  | **12** | 2 | 89.8 | 99.8 | 100 | 77.4 | 64.6 | 52.2 | |
|  |  |  | 4 | 3.2 | 47.6 | 82.2 |  |  |  | |
|  |  | **8** | 2 | 94.4 | 100 | 100 | 51.4 | 35.2 | 24.4 | |
|  |  |  | 4 | 14.2 | 66.4 | 88.2 |  |  |  | |
| **13 – 18 years** | | **24** | 2 | 84.2 | 99.2 | 100 | 96.6 | 92 | 90.6 | |
|  |  |  | 4 | 1.6 | 37 | 74.2 |  |  |  | |
|  |  | **12** | 2 | 90.4 | 100 | 100 | 73.2 | 61 | 54.6 | |
|  |  |  | 4 | 6.2 | 50 | 82.2 |  |  |  | |
|  |  | **8** | 2 | 95 | 100 | 100 | 48.2 | 36.2 | 24.6 | |
|  |  |  | 4 | 14.8 | 66.6 | 88 |  |  |  | |
|  | This table presents the probability of target attainment (PTA) for efficacy (C_max_/MIC ≥ 10, MIC of 2, and 4 mg/L) and safety (C_min_ < 5) for different dose intervals (every 24, 12, and 8 hr) across various paediatric age groups at dose of 10,15, and 20 mg/kg. | | | | | | | | |  |

Code S1. nlmixr2 code for the final amikacin PopPK model

The nlmixr2 code used to define the final amikacin PopPK model fitted to the combined neonatal and older paediatric dataset is provided below. The model includes a two-compartment structural model, allometric weight scaling, PMA maturation, age-adjusted serum creatinine, PNA maturation, concomitant glycopeptide use, inter-individual variability on CL, V1, Q and V2, and a combined residual error model.

```
final_amikacin_model <- function() {
  ini({
    tcl <- 1.72851276237038
    tv  <- 3.3789418101558
    tq  <- 0.0630953315121936
    tvp <- 3.40976623849171

    eta.cl + eta.v + eta.q + eta.vp ~ c(
      0.222597520852288,
      0.0186169355158569, 0.0506970970546489,
      0.229709539534124, 0.0211160875664682, 0.607283061694239,
      0.0334585338716289, 0.011002182666226, 0.0218988956792476,
      0.101563602367693
    )

    beta.wt.cl <- fix(0.75)
    beta.wt.v  <- fix(1)

    beta.cr.cl <- -0.72449
    beta.glycopeptide <- -0.20576

    hM <- c(0, 0.440873210259687, 1)
    hN <- c(0.01, 0.2839214638671)

    prop.err <- c(0, 0.24890419860156)
    add.err  <- c(0, 1.01995057448704)
  })

  model({
    maturation_pna <-
      hM + (1 - hM) * (1 - exp(-pna_days * hN))

    cl <- exp(
      tcl +
      eta.cl +
      beta.wt.cl * log_allo_wt +
      beta.cr.cl * log_allo_cr +
      beta.glycopeptide * glycopeptide
    ) * maturation * maturation_pna

    v <- exp(
      tv +
      eta.v +
      beta.wt.v * log_allo_wt
    )

    q <- exp(
      tq +
      eta.q +
      beta.wt.cl * log_allo_wt
    )

    vp <- exp(
      tvp +
      eta.vp +
      beta.wt.v * log_allo_wt
    )

    k   <- cl / v
    k12 <- q / v
    k21 <- q / vp

    d/dt(A1) = -k * A1 - k12 * A1 + k21 * A2
    d/dt(A2) =  k12 * A1 - k21 * A2

    cp = A1 / v

    cp ~ prop(prop.err) + add(add.err)
  })
}
```

Here, log_allo_wt represents the log-transformed allometric body-weight term, log_allo_cr represents the log-transformed ratio of observed serum creatinine to age-adjusted typical serum creatinine, maturation represents the PMA maturation function, pna_days is postnatal age in days, and glycopeptide indicates concomitant glycopeptide use.

1.	De Cock RF, Allegaert K, Schreuder MF et al. Maturation of the glomerular filtration rate in neonates, as reflected by amikacin clearance. *Clinical pharmacokinetics* 2012; **51**: 105-17.

2.	Sherwin CM, Svahn S, Van Der Linden A et al. Individualised dosing of amikacin in neonates: a pharmacokinetic/pharmacodynamic analysis. *Eur J Clin Pharmacol* 2009; **65**: 705-13.

3.	Allegaert K, Anderson BJ, Cossey V et al. Limited predictability of amikacin clearance in extreme premature neonates at birth. *Br J Clin Pharmacol* 2006; **61**: 39-48.

4.	Allegaert K, Scheers I, Cossey V et al. Covariates of amikacin clearance in neonates: the impact of postnatal age on predictability. *Drug Metabolism Letters* 2008; **2**: 286-9.

5.	Botha J, Du Preez M, Miller R et al. Determination of population pharmacokinetic parameters for amikacin in neonates using mixed-effect models. *Eur J Clin Pharmacol* 1998; **53**: 337-41.

6.	Illamola SM, Colom H, van Hasselt JC. Evaluating renal function and age as predictors of amikacin clearance in neonates: model‐based analysis and optimal dosing strategies. *Br J Clin Pharmacol* 2016; **82**: 793-805.

7.	Yu T, Stockmann C, Healy DP et al. Determination of optimal amikacin dosing regimens for pediatric patients with burn wound sepsis. *Journal of Burn Care & Research* 2015; **36**: e244-e52.

8.	Sherwin CM, Wead S, Stockmann C et al. Amikacin population pharmacokinetics among paediatric burn patients. *Burns* 2014; **40**: 311-8.
